# Concentration, Information, and Distributional Stability in High-Dimensional Portfolios: A Talagrand Stability Index Approach

**DOI:** 10.3390/e28070790

**Published:** 2026-07-12

**Authors:** Irina Georgescu, Jani Kinnunen

**Affiliations:** 1 Department of Economic Informatics and Cybernetics, Bucharest University of Economic Studies, Calea Dorobanți, 15-17, Sector 1, 010552 Bucharest, Romania; 2 Department of Information Systems, Åbo Akademi University, Tuomiokirkontori 3, 20500 Turku, Finland; jani.kinnunen@abo.fi

**Keywords:** high-dimensional portfolios, concentration inequalities, Wasserstein distance, Talagrand Stability Index, mutual information, portfolio diversification

## Abstract

This paper investigates the stability of high-dimensional financial portfolios using concentration inequalities, information-theoretic measures, optimal transport metrics, and financial network analysis. Asset returns are generated under both multivariate Gaussian and multivariate Student-t distributions. Equal Weight and Regularized Minimum Variance portfolios are evaluated across alternative portfolio dimensions. The results show that increasing portfolio dimension reduces portfolio risk, tail probabilities, and risk estimation errors, indicating stronger concentration and higher stability in high-dimensional settings. Entropy and mutual information measures reveal improved diversification and weaker dependence structures as portfolio size increases. To assess distributional robustness, a novel Talagrand Stability Index (TSI), combining Wasserstein distance and Kullback–Leibler divergence, is introduced. The results show that TSI decreases with portfolio dimension. Heavy-tailed Student-t returns generate weaker concentration effects, stronger dependence structures, and lower distributional stability than Gaussian returns. Mutual information-based financial networks reveal sparse and moderately interconnected dependence structures. To illustrate the practical applicability of the proposed framework, an empirical application based on daily returns of ten large U.S. equities during 2020–2025 is conducted, showing that the Regularized Minimum Variance portfolio achieves a marginally lower TSI than the Equal Weight portfolio. Robustness checks reported further indicate that this advantage is modest and outcome-dependent rather than decisive.

## 1. Introduction

High-dimensional financial systems have attracted increasing attention due to the rapid expansion of asset universes and the growing complexity of dependence structures in modern financial markets. While increasing the number of assets generally improves diversification opportunities, it also creates challenges related to estimation risk, portfolio stability, and nonlinear dependence. Traditional mean–variance frameworks often become unstable in high-dimensional settings because covariance estimation errors accumulate as dimensionality increases. Recent studies therefore emphasize the importance of robust optimization, regularization, and alternative dependence measures for improving portfolio stability and diversification [1,2].

At the same time, information-theoretic methods and optimal transport theory have emerged as powerful tools for analyzing uncertainty and dependence in financial systems. Measures such as Shannon entropy, mutual information, Kullback–Leibler divergence, and Wasserstein distance provide richer descriptions of financial dynamics than classical variance-based approaches because they capture nonlinear interactions and distributional geometry. In particular, mutual information has become increasingly relevant for portfolio optimization and financial network construction due to its ability to identify both linear and nonlinear dependence structures among assets [3].

Another development concerns the use of network-based representations of financial markets. Financial networks provide a structural perspective on systemic risk, interconnectedness, and shock transmission mechanisms by modeling assets as nodes connected through dependence relationships. Ref. [4] shows that mutual information networks reveal hidden topological structures that are not captured by standard correlation-based methods, including clustering effects, central nodes, and nonlinear information flows.

Motivated by these developments, this paper investigates the stability of high-dimensional financial portfolios by integrating concentration inequalities, entropy-based measures, optimal transport distances, and mutual information financial networks within a unified simulation framework. The analysis considers both multivariate Gaussian and multivariate Student-t return-generating processes, allowing the role of heavy-tailed distributions to be examined.

In addition, the paper introduces a Talagrand Stability Index (TSI) that combines Wasserstein distance and Kullback–Leibler divergence into a single measure of distributional robustness. By linking concentration of measure, transport-information inequalities, and network-based dependence structures, the study provides a comprehensive framework for evaluating portfolio stability in high-dimensional environments. A real-world application based on a diversified portfolio of major U.S. equities is presented to demonstrate the operational usefulness of the proposed TSI in practical portfolio evaluation and risk management.

## 2. Literature Review

### 2.1. High-Dimensional Portfolio Theory and Diversification

The foundations of portfolio theory were established by Harry Markowitz [5], who introduced the mean–variance framework as a tool for optimizing risk–return trade-offs. While very influential, this framework encounters significant limitations in high-dimensional settings, where the number of assets approaches or exceeds the sample size. In such contexts, estimation errors in the covariance matrix can lead to unstable portfolio allocations and poor out-of-sample performance.

Recent advances in high-dimensional finance emphasize the role of dimensionality in enhancing diversification and stability. Studies show that as the number of assets increases, idiosyncratic risks tend to cancel out, leading to more stable aggregate outcomes. This phenomenon is closely related to the concentration-of-measure principle, which has been extensively studied in probability theory and applied to financial systems. Increasing portfolio dimension leads to a systematic reduction in risk and improved estimation accuracy. Ref. [6] developed a measure-theoretic and distributionally robust portfolio optimization framework based on Wasserstein geometry, demonstrating that robust optimization methods provide stronger stability guarantees, improved finite-sample performance, and superior Sharpe ratios compared with traditional plug-in mean–variance approaches. Ref. [7] extended log-Sobolev and Talagrand inequalities by deriving new probabilistic error bounds and stability results, providing theoretical insights into concentration behavior and distributional convergence in high-dimensional optimization problems. Ref. [8] investigated the smoothness properties of entropic optimal transport and Wasserstein gradient flows, with theoretical results on stability, regularity, and displacement smoothness in entropy-based transport optimization frameworks.

Moreover, regularization techniques, such as shrinkage estimators, have been proposed to address instability in covariance estimation. These approaches improve numerical conditioning and enhance robustness in high-dimensional portfolio optimization, particularly in the presence of noisy data. Ref. [9] developed an optimal shrinkage estimator for high-dimensional mean–variance portfolio selection, demonstrating that incorporating estimation risk and random matrix theory significantly improves out-of-sample portfolio performance and robustness in large-dimensional financial systems. Ref. [10] proposed a shrinkage-based estimator for large covariance matrices, demonstrating that regularized covariance estimation substantially improves the stability and robustness of portfolio optimization in high-dimensional financial settings. Ref. [11] examined robust covariance estimators and Conditional Value-at-Risk constraints in portfolio optimization, showing that shrinkage covariance methods and clustering-based optimization improve portfolio stability, reduce drawdowns, and enhance out-of-sample performance under volatile market conditions. Ref. [12] proposed a neural network-based nonlinear shrinkage estimator for covariance matrices, demonstrating that integrating Ledoit–Wolf shrinkage with transformer-based learning improves minimum-variance portfolio optimization and reduces out-of-sample portfolio risk.

### 2.2. Information-Theoretic Approaches in Finance

Information theory has become an increasingly important tool in financial modeling, offering a flexible framework for capturing nonlinear dependencies and uncertainty. Measures such as Shannon entropy, mutual information (MI), and Kullback–Leibler (KL) divergence [13] provide deeper insights into the structure of financial data beyond traditional second-moment statistics.

Mutual information, in particular, has been widely used to quantify both linear and nonlinear dependence between financial variables. Unlike correlation, MI captures complex interactions and has been applied to portfolio diversification, risk management, and systemic risk analysis. The average mutual information between assets decreases with portfolio dimension, indicating a reduction in informational overlap and enhanced diversification.

The role of entropy-based portfolio optimization, where diversification is interpreted as maximizing informational uncertainty, is also highlighted in some studies. These approaches often outperform classical mean–variance methods, especially in volatile and high-dimensional environments. The integration of entropy and information-theoretic metrics thus provides a robust framework for understanding financial stability. Ref. [14] has questioned the adequacy of the traditional mean–variance framework, showing that entropy- and mutual information-based portfolio optimization models can better capture nonlinear dependence structures and improve diversification, stability, and extreme risk management in volatile financial markets. Ref. [15] proved that integrating Data Envelopment Analysis (DEA) with advanced optimization techniques such as mutual information (MI), Hilbert–Schmidt Independence Criterion (HSIC), and Maximum Mean Discrepancy (MMD) can improve portfolio diversification and risk-adjusted performance, offering a robust framework for modern portfolio management. Ref. [3] proposed a mean-entropy–mutual information portfolio optimization framework, showing that entropy-based models provide greater portfolio diversification and more stable performance under varying return constraints compared with traditional mean–variance approaches.

### 2.3. Optimal Transport and Distributional Metrics

In parallel with information-theoretic methods, optimal transport theory has emerged as a powerful tool for comparing probability distributions. The Wasserstein distance has gained particular attention due to its ability to capture geometric differences between distributions.

Unlike divergence-based measures such as KL, which focus on informational discrepancies, the Wasserstein distance accounts for the cost of transporting mass between distributions, providing a more intuitive notion of distance. This property makes it especially useful in financial applications, where the shape and support of return distributions are of critical importance.

Applications of optimal transport in finance include portfolio optimization, risk measurement, and model validation. Ref. [16] introduced the Wasserstein–Kelly portfolio optimization framework, demonstrating that distributionally robust optimization based on Wasserstein metrics can reduce estimation error, improve out-of-sample portfolio performance, and enhance the stability of growth-optimal investment strategies. Ref. [17] developed a robust portfolio optimization framework with stochastic dominance constraints based on Wasserstein distance, showing that robust portfolios can outperform non-robust approaches in out-of-sample evaluations while improving resilience to distributional uncertainty.

### 2.4. Concentration of Measure and Stability in High Dimensions

The concentration of measure concept is a fundamental property of high-dimensional systems. It states that, as dimensionality increases, many random variables become concentrated around their expected values, making large deviations progressively less likely [18,19]. Concentration inequalities provide exponential bounds on such deviations and represent a modern tool in probability theory and high-dimensional analysis [20].

In financial applications, concentration effects explain why larger portfolios often exhibit lower volatility and more stable aggregate returns. As the number of assets increases, asset-specific risks tend to diversify, reducing variability and improving portfolio robustness. Consequently, high-dimensional portfolios display stronger diversification benefits and higher stability than low-dimensional portfolios [21].

### 2.5. Talagrand Inequality and Transport–Information Relationships

A theoretical development linking optimal transport and information theory is the Talagrand inequality, introduced in [20]. This inequality establishes a relationship between the Wasserstein distance and the Kullback–Leibler divergence, showing that, under suitable conditions, geometric deviations between distributions can be bounded by their informational divergence.

This connection provides a powerful framework for analyzing stability in probabilistic systems, as it integrates two complementary perspectives: geometry and information. Building on this idea, recent research has explored transport–entropy inequalities in various applications on statistical learning, stochastic processes, and financial modeling.

Ref. [22] developed a distributionally robust portfolio allocation framework based on Wasserstein-distance optimization and behavioral utility functions, demonstrating that simultaneously accounting for market uncertainty and investor irrationality can improve investment returns while reducing portfolio risk. Ref. [23] proposed a data-driven distributionally robust Kelly portfolio optimization framework based on coherent Wasserstein metrics, showing that the model improves portfolio stability, controls volatility and maximum drawdown, and outperforms classical Kelly and type-1 Wasserstein–Kelly approaches. Ref. [24] proposed a Wasserstein generative modeling framework for robust portfolio optimization, proving that combining Wasserstein-based distributionally robust optimization with generative adversarial learning improves Sharpe ratios, reduces maximum drawdowns, and enhances portfolio stability under distributional uncertainty and volatile market conditions. Ref. [25] extended Talagrand’s transportation inequality by introducing a constrained formulation linking Kullback–Leibler divergence and Wasserstein-2 distance, providing a theoretical framework for analyzing distributional stability and probabilistic transport relationships.

### 2.6. Financial Networks and Dependence Structures

Network-based approaches have become increasingly applied in the analysis of financial systems, providing a structural perspective on interdependencies between assets. In such frameworks, assets are represented as nodes, while edges capture relationships such as correlations or information flows.

Recent studies emphasize the importance of nonlinear dependence measures, such as mutual information, in constructing financial networks. These networks reveal hidden structures that are not captured by linear correlation-based methods, including clustering, community formation, and systemic importance. Ref. [26] included transfer entropy constraints into portfolio optimization, showing that entropy-based dependency measures can improve portfolio diversification, reduce systemic risk, and enhance stability relative to conventional risk-management approaches such as Value at Risk and Conditional Value-at-Risk. Ref. [27] proposed an automated decision-support framework for portfolio allocation that combines mutual information, entropy weighting, and multi-criteria decision-making techniques, demonstrating improved loss control and robust portfolio performance during market downturns. Ref. [4] developed a nonlinear mutual information network framework with global motion filtering, showing that portfolios constructed from filtered mutual information networks achieve higher Sharpe and Sortino ratios and remain robust across bullish, bearish, and turbulent market conditions. Ref. [28] applied Shannon entropy and mutual information to examine financial market efficiency under speculative bubbles, showing that expansionary monetary policy and credit growth can intensify market inefficiencies and nonlinear dependencies in financial systems.

### 2.7. Research Gap and Contribution

Despite significant advances in high-dimensional finance, information theory, and optimal transport, the integration of these frameworks into a unified measure of financial stability remains limited. Existing studies focus on individual metrics, such as entropy, Wasserstein distance, or KL divergence, without capturing their joint behavior.

This paper contributes to the literature by introducing a novel stability measure—the Talagrand Stability Index—that combines geometric and informational perspectives into a single framework. By linking concentration of measure, information theory, and network analysis, the study provides a comprehensive approach to understanding stability in high-dimensional financial systems.

## 3. Theoretical Framework

**Figure 1:**
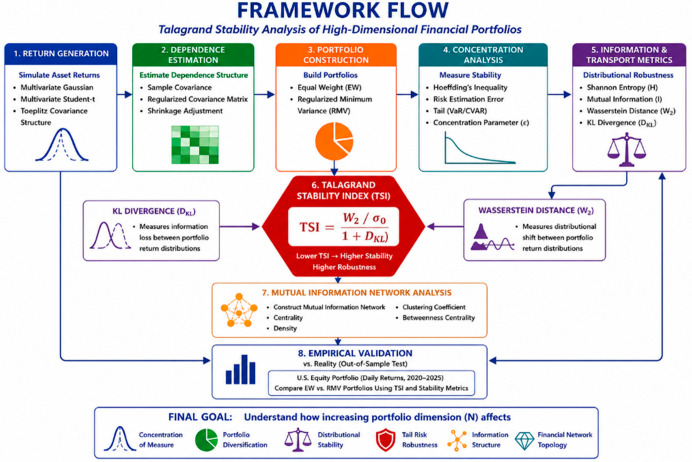
Methodological flow.

The methodological flow follows the steps of Figure 1:

To analyze the stability of high-dimensional financial portfolios, we consider a market composed of N risky assets. Let

(1) $$R_{t}=({R_{1t},\ldots R_{Nt})}^{T}$$

denote the vector of asset returns at time t, where $R_{it}$ represents the return of asset i at time t. The return vector is characterized by a mean vector $\mu =E[R_{t}]$ and a covariance matrix $\Sigma =Cov(R_{t})$. To generate structured dependence across assets, the covariance matrix is specified using a Toeplitz structure $\Sigma_{ij}=\sigma^{2}\rho^{\left|i-j\right|}$, where $\sigma^{2}$ denotes the variance of returns, ρ∈(0,1) is a correlation decay parameter, and |i−j| represents the absolute difference between the indices of assets *i* and *j*. The correlation decay parameter ρ controls how quickly pairwise correlations decrease as the distance between assets increases in the Toeplitz covariance matrix. This specification implies that correlations decay exponentially as the distance between asset indices increases. Such Toeplitz covariance structures are widely used in high-dimensional simulation studies due to their tractability and positive definiteness [29].

Asset returns are generated from a multivariate Gaussian distribution $R_{t}\sim N(\mu ,\Sigma )$, where μ denotes the vector of expected returns and Σ represents the covariance matrix of asset returns.

The Gaussian distribution serves as the classical benchmark model in portfolio theory [5]. Asset returns are also generated from a multivariate Student-t distribution $R_{t}\sim t_{\nu }(\mu ,\Sigma )$. The Student-t distribution allows for heavier tails than the Gaussian distribution and therefore captures a higher probability of extreme return realizations. This feature makes it suitable for modeling financial returns characterized by tail risk and excess kurtosis.

### 3.1. Portfolio Construction

Let $w={\left(w_{1},w_{2},\ldots ,w_{N}\right)}^{T}$ denote the portfolio weight vector, where $w_{i}$ represents the proportion of total capital invested in asset *i*. The weights satisfy the standard budget constraint

$$\sum_{i=1}^{N}w_{i}=1.$$

Let $R_{p}$ denote the random portfolio return, while $R_{p,t}$ denotes its realization at time *t*.

The portfolio return at time t is defined as

(2) $$R_{p,t}=w^{T}R_{t}.$$

The theoretical variance of the portfolio return is given by

(3) $$\sigma_{p}^{2}=w^{T}\Sigma w$$

Σ denotes the covariance matrix of asset returns. The corresponding theoretical portfolio risk is:

(4) $$\sigma_{P}=\sqrt{w^{T}\Sigma w}$$

Two portfolio construction strategies are considered.

The first strategy is the Equal Weight portfolio, in which all assets receive identical weights. In this case $w_{i}=\frac{1}{N},\;i=1,\ldots ,N$. This allocation rule provides a simple diversification benchmark and avoids estimation error in the covariance matrix [30].

The second strategy is a Regularized Minimum Variance portfolio. The classical minimum-variance problem seeks to minimize portfolio variance

(5) $$\underset{w}{\min}\;w^{T}Sw$$

subject to the constraint $1^{T}w=1$, where *S* denotes the sample covariance matrix estimated from the data, and ***1*** is a vector of ones.

However, in high-dimensional settings, the covariance estimator can become unstable [31]. To address this issue, a regularization term is introduced, leading to the optimization problem

(6) $$\underset{w}{\min}\left(w^{T}Sw+\lambda {\left\|w\right\|}^{2}\right)$$

Subject to $1^{T}w=1$. Here λ>0 is a regularization parameter controlling the degree of shrinkage, and ${\left\|w\right\|}^{2}=w^{T}w$ represents the squared Euclidean norm of the weight vector. The optimization corresponds to a ridge-Regularized Minimum Variance problem, in which the covariance matrix is regularized by adding λI, following the classical ridge regression approach of [32]. The resulting portfolio weights are

(7) $$w=\frac{{\left(S+\lambda I\right)}^{-1}1}{1^{T}{\left(S+\lambda I\right)}^{-1}1}$$

where *I* is the identity matrix.

Regularization stabilizes covariance matrix estimation by shrinking the sample covariance matrix toward a more stable covariance estimator, thereby reducing estimation error and producing a more stable inverse covariance matrix. This is important in high-dimensional settings, where the sample covariance matrix is often ill-conditioned or singular, leading to unstable portfolio weights [31].

Portfolio performance is evaluated using Monte Carlo simulations. For each portfolio dimension N, simulated return series are generated using the Gaussian data-generating process described above. For the heavy-tailed scenario, returns are generated from a Student-t distribution with ν=5 degrees of freedom throughout all Monte Carlo simulation experiments. The analysis considers several values of N representing increasing portfolio dimensionality.

Since portfolio returns are continuous random variables, the empirical and theoretical return distributions are discretized using histograms with 50 equally spaced bins defined over the common support of both distributions. The resulting empirical probability masses are then used to estimate the Kullback–Leibler divergence. To avoid numerical instability arising from zero probabilities, a small constant 10^−10^ is added before normalization.

For each simulation run, the sample is divided into two parts. The first part, containing $T_{in}$ observations, is used to estimate the covariance matrix and construct portfolio weights. The second part, consisting of $T_{out}$ observations, is used to evaluate the out-of-sample performance of the portfolio. This procedure is repeated across multiple Monte Carlo replications in order to assess the stability and robustness of the results.

### 3.2. Simulation Parameters

The simulation framework relies on a set of parameters governing the data-generating process, portfolio construction, and evaluation procedures.

The number of assets in the portfolio is denoted by N, which varies over the set $N\in \left\{10,25,50,75,100,150,200\right\}$, allowing the analysis to capture the transition from low-dimensional to high-dimensional portfolio settings. The sample is divided into an in-sample period of length $T_{in}=250\;$observations and an out-of-sample period of length $T_{out}=250\;$corresponding approximately to one trading year each. The correlation decay parameter was set to ρ=0.4, representing a moderate dependence structure among assets. This value is consistent with simulation studies based on Toeplitz covariance matrices and AR(1)-type correlation structures frequently used in high-dimensional covariance estimation and portfolio optimization research [29,31]. A moderate value of ρ allows meaningful dependence between assets while preserving diversification effects and concentration phenomena.

The variance parameter is set to $\sigma^{2}=0.0004$, while the expected return of each asset is assumed to be constant and equal to $\mu_{i}=0.0005$, reflecting realistic daily return dynamics.

The regularization parameter used in the minimum-variance portfolio is set to λ=0.10, controlling the degree of shrinkage applied to portfolio weights and improving numerical stability in high-dimensional settings [31].

The Monte Carlo simulation is repeated B = 200 times for each value of N, ensuring statistical robustness of the results. To evaluate concentration properties, a grid of deviation thresholds is defined as ε∈[0.001, 0.08], consisting of 30 equally spaced values. These thresholds are used to estimate empirical tail probabilities and the concentration parameter.

### 3.3. Portfolio Performance Metrics

The realized mean return of the portfolio is computed as:

(8) $${\hat{\mu }}_{p}=\frac{1}{T}\sum_{t=1}^{T}R_{p,\;t}$$

Portfolio risk is measured by the realized standard deviation

(9) $${\hat{\sigma }}_{p}=\sqrt{\frac{1}{T-1}\sum_{t=1}^{T}{\left(R_{p,t}-{\hat{\mu }}_{p}\right)}^{2}}$$

The absolute difference between realized and theoretical risk is defined as

(10) $$E_{\sigma }=\left|{\hat{\sigma }}_{p}-\sigma_{p}\right|$$

Across the Monte Carlo replications, the average realized portfolio risk is computed as:

(11) $${\bar{\hat{\sigma }}}_{p}=\frac{1}{B}\sum_{b=1}^{B}{\hat{\sigma }}_{p}^{\left(b\right)}$$

while the average risk error is given by:

(12) $${\bar{E}}_{\sigma }=\frac{1}{B}\sum_{b=1}^{B}E_{\sigma }^{\left(b\right)}$$

Portfolio diversification is evaluated using the Herfindahl–Hirschman index [33,34]:

(13) $$HHI=\sum_{i=1}^{N}w_{i}^{2}$$

Lower HHI values indicate stronger diversification because portfolio weights are distributed more evenly across assets, whereas higher HHI values indicate greater concentration in a smaller number of holdings.

### 3.4. Concentration of Portfolio Returns

To analyze the stability of portfolio returns, we examine the probability that the portfolio return deviates from its expected value by more than a threshold ε:

(14) $$P(\left|R_{p}-E\left[R_{p}\right]\right|>\varepsilon )$$

In the Monte Carlo framework considered in this paper, portfolio returns are generated as independent and identically distributed observations from either a multivariate Gaussian distribution or a multivariate Student-t distribution with a fixed covariance matrix. For the Gaussian case, classical concentration inequalities motivate an exponential upper bound for the tail probability of portfolio returns of the form:

(15) $$P(\left|R_{p}-E\left[R_{p}\right]\right|>\varepsilon )\leq 2e^{-c\varepsilon^{2}}$$

The parameter c represents the concentration rate, which measures how rapidly the tail probability decreases as the deviation threshold increases. For the Gaussian simulations, the exponential form is motivated by classical concentration inequalities. For the Student-t simulations, the same functional form is used as an empirical model for estimating the decay rate of tail probabilities rather than as a formal concentration inequality [35,36].

Empirically, the concentration parameter is estimated using the regression model

(16) $$logP\left(\left|R_{p}-E\left[R_{p}\right]\right|>\varepsilon \right)=a-c\varepsilon^{2}$$

Although the classical concentration inequalities motivating this specification are primarily associated with sub-Gaussian probability measures, the Student-t distribution is included in this study as a heavy-tailed benchmark to assess the empirical robustness of the proposed stability measures under departures from the theoretical assumptions. Consequently, the concentration analysis for Student-t returns should be interpreted as an empirical comparison rather than as a theoretical validation of the underlying concentration inequalities.

The empirical concentration parameter is therefore estimated separately under Gaussian and Student-t data-generating processes and subsequently compared to assess the effect of heavy tails on portfolio stability.

### 3.5. Distributional Stability Measures

To evaluate distributional stability beyond variance, the Wasserstein distance between the empirical distribution of portfolio returns and a reference distribution is computed. To quantify the distributional stability of portfolio returns, we compare the empirical portfolio return distribution with a theoretical return distribution. Let P denote the empirical probability distribution of portfolio returns obtained from a single Monte Carlo replication (or from the empirical portfolio returns in the real-data application). Let Q denote the corresponding theoretical reference distribution implied by the assumed data-generating process. Specifically, in the Gaussian experiments, Q is the normal distribution with portfolio mean $\mu_{P}$ and the standard deviation $\sigma_{P}$. In the heavy-tailed experiments, Q is the corresponding Student-t distribution with identical location and scale parameters and the specified degrees of freedom. The parameters of the reference distribution are determined from the theoretical portfolio moments in the Monte Carlo experiments and from the estimated portfolio moments in the empirical application.

The Wasserstein distance of order p≥1 between two probability measures P and Q on **R**, where P denotes the empirical portfolio return distribution and Q the corresponding theoretical Gaussian or Student-t reference distribution can be expressed through their quantile functions as [37]:

(17) $$W_{p}(P,Q)=\int_{0}^{1}{\left|F_{P}^{-1}\left(u\right)-F_{Q}^{-1}(u)\right|}^{p}du$$

The 2-Wasserstein distance is defined as:

(18) $$W_{2}\left(P,Q\right)={\left(\int_{0}^{1}{\left(F_{P}^{-1}\left(u\right)-F_{Q}^{-1}(u)\right)}^{2}du\right)}^{1/2}$$

This metric measures the minimal transportation cost required to transform one distribution into another [38].

Portfolio diversification is further assessed using Shannon entropy:

(19) $$H\left(w\right)=-\sum_{i=1}^{N}w_{i}logw_{i}$$

and its normalized version $H^{*}=\frac{H(w)}{\log N}$.

Higher entropy values indicate more evenly distributed portfolio weights.

To further investigate the relationship between distributional deviations and information divergence, we introduce a stability indicator inspired by Talagrand-type transport inequalities. Ref. [20] established a fundamental connection between optimal transport distances and entropy, showing that for suitable probability measures:

(20) $$W_{2}^{2}\left(P,Q\right)\leq C\cdot KL(P∥Q)$$

where $W_{2}^{2}\left(P,Q\right)$ denotes the squared 2-Wasserstein distance between probability measures P and Q. KL(P∥Q) is the Kullback–Leibler divergence [13], C>0 is a constant. Motivated by this relationship, we construct an empirical Talagrand Stability Index (TSI) defined as

(21) $$TSI(P,Q)=\frac{W_{2}^{2}(P,Q)}{KL(P||Q)}$$

From a financial perspective, TSI can be interpreted as a measure of the robustness of portfolio returns, capturing the extent to which deviations from the theoretical distribution are driven by structural distortions rather than informational fluctuations. Lower values of TSI indicate that deviations from the reference distribution are small relative to their informational complexity, suggesting greater stability of portfolio returns.

TSI is asymmetric since the Kullback–Leibler divergence is not symmetric. In the present framework, this asymmetry reflects the directional nature of the comparison. The empirical portfolio return distribution P represents the observed portfolio behavior, whereas the reference distribution Q represents the benchmark implied by the assumed return-generating model. Consequently, TSI(P,Q) quantifies how robust the observed portfolio is relative to the benchmark. Reversing the order to TSI(Q,P) would instead evaluate how well the benchmark approximates the empirical distribution, addressing a different financial question rather than the robustness of the observed portfolio.

The proposed TSI is based on the squared 2-Wasserstein distance because Talagrand’s classical transport-information inequality is formulated in terms of $W_{2}\left(P,Q\right)$, providing the theoretical foundation for the index.

#### Theoretical Properties of the Talagrand Stability Index

We can prove some properties of TSI as follows.

**Proposition 1** (Non-negativity)**.** *For any probability distributions* P *and* Q *satisfying* $KL(P|\left|Q\right)>0$*, TSI satisfies* $TSI\left(P,Q\right)\geq 0$.

**Proof.**  Since $W_{2}^{2}\left(P,Q\right)\geq 0$ and $KL(P|\left|Q\right)\geq 0$, it follows that $TSI\left(P,Q\right)\geq 0$. □

**Proposition 2**  
(Talagrand Upper Bound)**.** *Suppose that* Q *satisfies Talagrand’s transport-information inequality*

$$W_{2}^{2}\left(P,Q\right)\leq C\cdot KL(P∥Q)$$

*for some constant C > 0. Then* $TSI\left(P,Q\right)\leq C$.

**Proof.**  From Talagrand’s inequality,

$$W_{2}^{2}\left(P,Q\right)\leq C\cdot KL(P∥Q)$$

Dividing both sides by KL(P∥Q), it follows that $TSI\left(P,Q\right)\leq C$. □

This result establishes a direct theoretical connection between the proposed index and Talagrand’s transportation inequality. The index can therefore be interpreted as a normalized transport-information coefficient.

**Proposition 3**  
(Monotonicity with respect to Wasserstein distance)**.** *For fixed informational divergence* $KL\left(P∥Q\right)=K>0$*, the Talagrand Stability Index is strictly increasing in the 2-Wasserstein distance*  $W_{2}\left(P,Q\right)$.

**Proof.**  Let $f\left(W_{2}\right)=\frac{{W_{2}}^{2}}{K}$. Differentiating with respect to $W_{2}$, $\frac{df}{dW_{2}}=\frac{2W_{2}}{K}$. Since $K>0,\;W_{2}>0$, it follows that $\frac{df}{dW_{2}}>0$.

Therefore, TSI increases monotonically with the Wasserstein distance. □

**Proposition 4**  
(Monotonicity with respect to information divergence)**.** *For fixed Wasserstein distance* $W_{2}\left(P,Q\right)=W_{2}>0$*, the Talagrand Stability Index decreases as informational divergence increases.*

**Proof.**  Let $g\left(K\right)=\frac{{W_{2}}^{2}}{K}$. Differentiating with respect to K, $\frac{dg}{dK}=\frac{-{W_{2}}^{2}}{K^{2}}<0$. Thus, TSI is strictly decreasing in $KL\left(P∥Q\right)$. □

**Proposition 5**  
(Distributional convergence)**.** *Let* $\left\{P_{n}\right\}$ *be a sequence of probability measures such that* $W_{2}(P_{n},Q)\to 0$*. Then* $TSI(P_{n},Q)\to 0$*, provided that* $KL\left(P_{n}∥Q\right)$ *does not converge to 0 faster than* $W^{2}(P_{n},Q)$.

**Proof.**  By definition, $TSI\left(P_{n},Q\right)=\frac{W_{2}^{2}(P_{n},Q)}{KL(P_{n}||Q)}$. Since $W_{2}\left(P_{n},Q\right)\to 0$, it follows that $W_{2}^{2}\left(P_{n},Q\right)\to 0$. If $KL\left(P_{n}∥Q\right)$ does not converge to zero faster than $W_{2}^{2}\left(P_{n},Q\right)$, then $TSI\left(P_{n},Q\right)\to 0$. □

**Remark 1.** 
*This theoretical framework assumes independent and identically distributed portfolio returns. For non-stationary or time-dependent return-generating processes, TSI is expected to become larger and more variable, reflecting reduced distributional stability.*

### 3.6. Mutual Information Financial Network Construction

To further investigate the nonlinear dependence structure among financial assets, we construct a financial network based on mutual information (MI). In this framework, each asset is represented as a node, while edges capture nonlinear dependence relationships between asset returns. Unlike Pearson correlation, which measures only linear dependence, mutual information captures both linear and nonlinear interactions and is therefore suitable for modeling complex financial systems characterized by nonlinear dynamics and hidden interdependencies [39].

The mutual information between two random variables $X_{i}$ and $X_{j}$ is:

(22) $${MI}_{ij}=MI\left(X_{i},X_{j}\right)=\sum_{x_{i},x_{j}}p\left(x_{i},x_{j}\right)log(\frac{p\left(x_{i},x_{j}\right)}{p\left(x_{i}\right)p(x_{j})})$$

where $p\left(x_{i},x_{j}\right)$ denotes the joint probability distribution of $X_{i}$ and $X_{j}$, while $p\left(x_{i}\right)$ and $p(x_{j})$ represent the corresponding marginal distributions. Mutual information measures the amount of shared information between two variables and takes nonnegative values, with larger values indicating stronger dependence structures [39].

To estimate mutual information empirically, continuous asset returns are discretized into 10 equal-frequency bins using the infotheo package in R Studio 2023.12.1 Build 402 prior to constructing the mutual information matrix. The resulting discretized observations are then used to estimate the pairwise mutual information values, producing a matrix that summarizes the nonlinear dependence structure among all assets in the portfolio. The resulting matrix provides pairwise measures of nonlinear dependence among all assets in the portfolio.

Since fully connected networks are difficult to interpret and may contain noise, a filtering procedure is applied to retain only the strongest dependence relationships. Namely, a quantile-based threshold is employed:

(23) $$\tau =Q_{0.90}({MI}_{ij})$$

where $Q_{0.90}(.)$ denotes the 90th percentile of the upper triangular elements of the mutual information matrix. Thus, only the strongest 10% of nonlinear dependence relationships are preserved in the network. The use of a high quantile threshold reduces noise and prevents the network from becoming too dense [40].

Based on this threshold, the weighted adjacency matrix $A_{ij}\;$of the financial network is constructed as follows:

(24) $$A_{ij}=\left\{\begin{array}{cc} {MI}_{ij}, & {MI}_{ij}\geq \tau \;\\ 0, & {MI}_{ij}<\tau \; \end{array}\right.$$

The resulting graph is therefore a sparse weighted mutual information network in which edge weights reflect the intensity of nonlinear dependence between asset returns.

To characterize the structural properties of the network, several graph-theoretic indicators are computed following [40,41].

Edge density, which measures the proportion of realized connections relative to all possible connections in the network G:

(25) $$Density\left(G\right)=\frac{2E}{N(N-1)}$$

E denotes the number of edges, and N represents the number of nodes. Higher density values indicate stronger global interconnectedness among financial assets.

The average degree is defined as:

(26) $$\bar{k}=\frac{1}{N}\sum_{i=1}^{N}k_{i}$$

$k_{i}$ denotes the degree of node i. The average degree measures the average number of direct connections associated with each asset in the network.

To evaluate local clustering and community formation, the transitivity or clustering coefficient is computed:

(27) $$C_{clust}=\frac{3*Number\;of\;triangles}{Number\;of\;connected\;triplets}$$

This measure captures the tendency of neighboring nodes to form interconnected groups. Higher clustering coefficients indicate stronger local cohesion and more pronounced clustering structures within the financial system [40].

Finally, the systemic importance of nodes is evaluated using betweenness centrality:

(28) $$BC\left(v\right)=\sum_{s\neq v\neq t}\frac{\sigma_{st}(v)}{\sigma_{st}}$$

where $\sigma_{st}$ denotes the number of shortest paths between nodes s and t, while $\sigma_{st}(v)$ represents the number of those paths passing through node v. Nodes with high betweenness centrality play an intermediary role in the transmission of information and shocks across the financial network [41].

## 4. Empirical Results

### 4.1. Portfolio Risk, Diversification, and Concentration

Table 1 presents the evolution of portfolio risk, its stability across simulations, the risk estimation error, and diversification under both Gaussian and t-return-generating processes. First, portfolio risk declines monotonically as the number of assets increases. Under Gaussian returns, average realized risk decreases from 0.00920 for N = 10 assets to 0.00216 for N = 200, representing a reduction of approximately 76.5%. A similar pattern is observed under Student-t returns, where average risk falls from 0.01180 to 0.00275.

Second, portfolios generated from Student-t returns are riskier than their Gaussian counterparts. For example, at N = 10, the average risk under the Student-t distribution is 0.01180 compared with 0.00920 under the Gaussian distribution. At N = 200, Student-t portfolios remain more volatile (0.00275 versus 0.00216). On average, Student-t portfolios exhibit about 29% higher realized risk, reflecting the impact of heavy tails and a greater probability of extreme return realizations.

Third, risk stability improves with diversification. The standard deviation of realized risk decreases as N increases. Under Gaussian returns, SD Risk declines from 0.000422 to 0.000100, while under Student-t returns, it decreases from 0.000899 to 0.000211. The Student-t process exhibits larger variability at all dimensions. Heavy-tailed distributions generate less stable portfolio outcomes. This behavior is explained by the statistical properties of heavy-tailed distributions. Compared with the Gaussian distribution, heavy-tailed distributions assign a higher probability to extreme return realizations. These rare but large observations increase the variability of individual asset returns and contribute disproportionately to portfolio volatility. Although diversification continues to reduce idiosyncratic risk, extreme observations are not fully diversified away, leading to higher realized portfolio risk, larger estimation errors, and weaker concentration effects.

Fourth, risk estimation errors decline with portfolio size. Diversification improves the accuracy of risk assessment. The average risk error falls from 0.000340 to 0.000077 under Gaussian returns and from 0.000701 to 0.000170 under Student-t returns. The larger errors observed under Student-t returns highlight the challenges associated with modeling and forecasting risk in environments characterized by extreme events.

Finally, HHI decreases from 0.1000 to 0.0050 as N increases. Since equal portfolio weights are employed, the HHI values are identical for the two distributions and depend only on N.

Table 2 reports the estimated concentration parameter c obtained from the exponential decay model of tail probabilities, along with the corresponding intercepts, or both Gaussian and Student-t return-generating processes. The parameter c measures the rate at which the probability of deviations from the expected portfolio return decreases as the threshold ε increases. A central finding is the monotonic increase in the concentration parameter c as the portfolio dimension N increases. Under the Gaussian distribution, the concentration parameter rises from 0.676 for (N = 10) to more than 11.5 for (N = 200), revealing a pronounced concentration-of-measure effect. A similar upward trend is observed under the Student-t distribution. The estimated concentration parameters remain lower, increasing from 0.117 to 0.798. The presence of heavy tails weakens the concentration effect by increasing the likelihood of extreme return realizations.

This pattern indicates that the tail probability

$$P(\left|R_{p}-E\left[R_{p}\right]\right|>\varepsilon )$$

decays significantly faster in higher-dimensional portfolios. In other words, large deviations from the mean become exponentially less likely as N increases.

This result provides empirical evidence of a concentration-of-measure phenomenon in financial portfolios. As N grows, the aggregation of asset returns leads to stabler outcomes. Portfolio returns are concentrated around their expected value. This behavior is consistent with theoretical predictions from high-dimensional probability, where sums or averages of many weakly dependent variables have reduced variability and stronger concentration.

Another observation is that the estimates of c are nearly identical for the Equal Weight and Regularized Minimum Variance portfolios for all values of N and for both return-generating processes. The differences between the two strategies are negligible. This finding suggests that, under the symmetric covariance structure and homogeneous expected returns assumed in the model, the optimization procedure does not alter the concentration properties of portfolio returns. Instead, both portfolio strategies converge to similar weight allocations, having identical probabilistic behavior.

The intercept term remains stable in each distributional framework. For Gaussian returns, the intercept values range between −0.8 and −0.5, while for Student-t returns, they range between −4.6 and −2. This stability indicates that the baseline level of tail probabilities is higher under heavy-tailed return distributions.

These results prove that increasing the number of assets in a portfolio not only reduces risk but also improves the probabilistic stability of returns by strengthening concentration effects. The Gaussian and Student-t distributions represent benchmark cases for light-tailed and heavy-tailed return dynamics. If asset returns followed even heavier-tailed distributions, concentration effects would be expected to weaken further because extreme observations would occur more frequently. Consequently, portfolio risk, risk estimation uncertainty, and distributional instability would likely increase.

**Figure 2:**
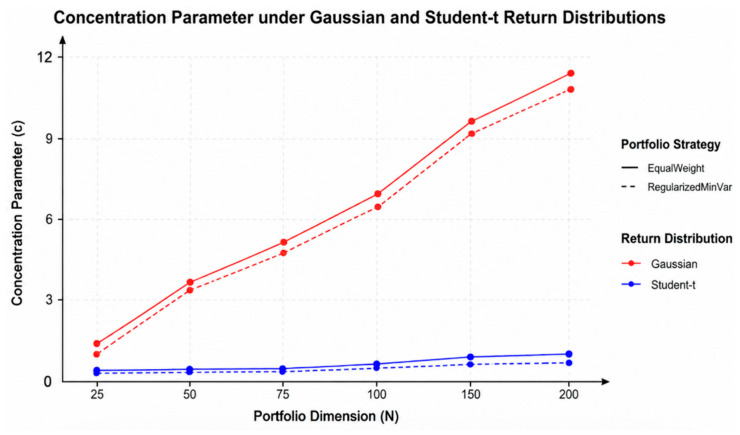
Concentration of portfolio returns in high dimensions: estimated exponential decay parameter.

Figure 2 illustrates the evolution of the concentration parameter (c) with portfolio dimension under Gaussian and Student-t return distributions. The concentration effect strengthens as the number of assets increases, indicating greater stability of portfolio returns in larger portfolios. While both distributions exhibit the same qualitative pattern, concentration is weaker under Student-t returns. This highlights the impact of heavy tails on portfolio stability. The nearly overlapping curves for the two portfolio strategies suggest that the distributional assumption is more important than the allocation rule in determining concentration behavior.

To formally assess the relationship between portfolio dimension and concentration, we estimate a linear model in Table 3, where the concentration parameter is regressed on the number of assets. The results indicate a positive and statistically significant relationship (β = 0.059, *p* < 0.001), with an R^2^ of 0.993. This confirms that concentration effects increase systematically with portfolio dimension in the case of a Gaussian distribution.

Table 4 reports the linear relationship between portfolio dimension and the concentration parameter under the Student-t distribution. The estimated coefficient for portfolio dimension is positive and statistically significant (β = 0.004, *p* < 0.001), indicating that concentration effects strengthen as N increases. However, the magnitude of the effect is considerably smaller than under the Gaussian distribution, suggesting that heavy-tailed returns weaken the concentration phenomenon.

**Figure 3:**
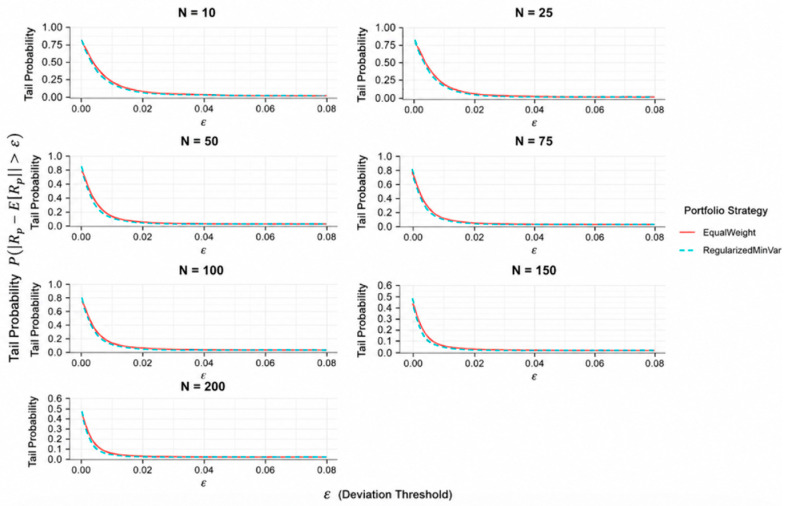
Empirical tail probability decay across portfolio dimensions—Gaussian distribution.

Figure 3 shows the empirical tail probabilities $P(\left|R_{p}-E\left[R_{p}\right]\right|>\varepsilon )$ for Gaussian portfolio returns across different portfolio dimensions. The horizontal axis represents the deviation threshold ε, while the vertical axis reports the corresponding empirical tail probabilities. As ε increases, the probability of observing large deviations decreases rapidly. This rapid decay indicates a stronger concentration of portfolio returns and greater portfolio stability as the portfolio dimension increases. The near overlap of the Equal Weight and Regularized Minimum Variance portfolios suggests similar tail behavior under the symmetric setting considered. These findings are consistent with the portfolio dimensionality framework of [42] and the high-dimensional perspective of stochastic portfolio theory developed by [43].

**Figure 4:**
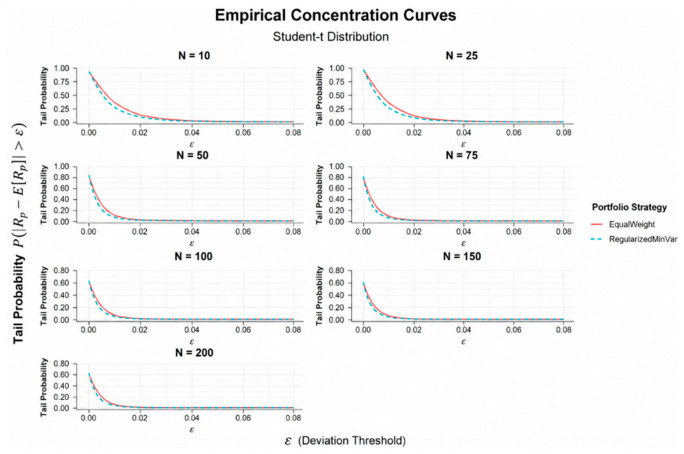
Empirical tail probability decay across portfolio dimensions—Student-t distribution.

Figure 4 presents the empirical tail probabilities $P(\left|R_{p}-E\left[R_{p}\right]\right|>\varepsilon )$ under the Student-t distribution. The horizontal axis represents the deviation threshold ε, while the vertical axis reports the corresponding empirical tail probabilities. As ε increases, the probability of observing large deviations decreases, although more gradually than in the Gaussian case because of the heavier tails of the Student-t distribution. The probability of large deviations becomes smaller as the portfolio dimension increases, indicating better concentration and greater portfolio stability through diversification. The near overlap of the Equal Weight and Regularized Minimum Variance portfolios suggests that the choice of portfolio allocation rule has only a limited effect on the concentration behavior under the simulation setting considered.

**Figure 5:**
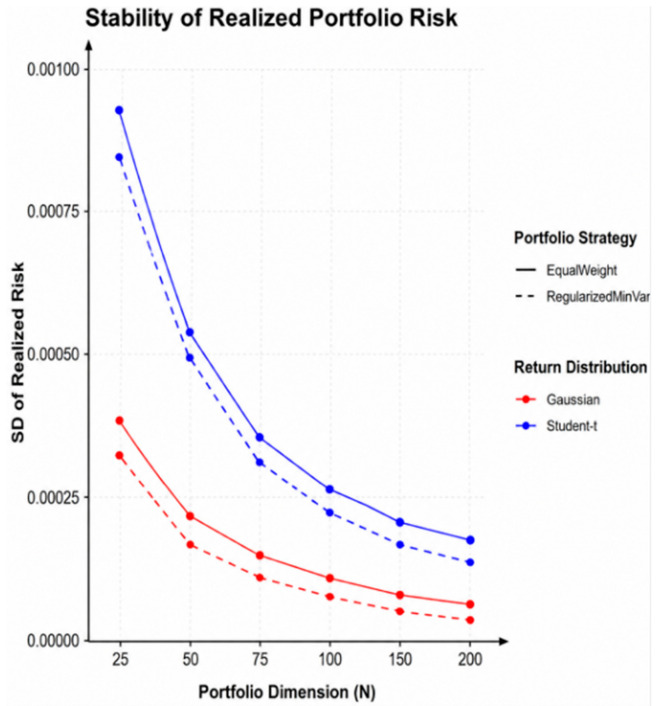
Stability of realized portfolio risk across dimensions.

Figure 5 reports the standard deviation of the realized portfolio risk across the Monte Carlo replications:

$$SD\left({\hat{\sigma }}_{p}\right)=\sqrt{\frac{1}{B-1}\sum_{b=1}^{B}{\left({\hat{\sigma }}_{p}^{\left(b\right)}-{\bar{\hat{\sigma }}}_{p}\right)}^{2}}$$

where ${\hat{\sigma }}_{p}^{\left(b\right)}$ denotes the realized portfolio risk in Monte Carlo replication b, and ${\bar{\hat{\sigma }}}_{p}\;$is the average realized portfolio risk across all replications. The figure shows that this variability decreases as the number of assets increases under both Gaussian and Student-t return distributions.

This indicates that portfolio risk becomes more stable and predictable in higher dimensions, highlighting the benefits of diversification. Risk variability remains higher under the Student-t distribution, reflecting the influence of heavy tails and a greater likelihood of extreme outcomes. The nearly overlapping portfolio strategies suggest that the return distribution has a stronger effect on risk stability than the allocation rule. These findings are consistent with [44], who shows that portfolio risk decreases as portfolio size increases and tends to stabilize beyond a certain threshold, reflecting diminishing marginal diversification benefits and improved robustness of portfolio outcomes.

**Figure 6:**
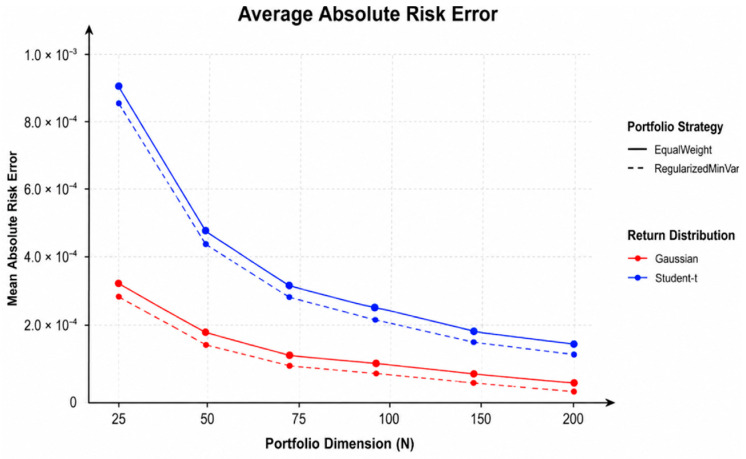
Average absolute risk error across portfolio dimensions.

Figure 6 reports the mean absolute risk error across B Monte Carlo replications:

(29) $${\bar{E}}_{\sigma }=\frac{1}{B}\sum_{b=1}^{B}\left|{\hat{\sigma }}_{p}^{\left(b\right)}-\sigma_{p}\right|$$

where ${\hat{\sigma }}_{p}^{\left(b\right)}$ denotes the realized portfolio risk in Monte Carlo replication b, and $\sigma_{p}$ is the corresponding theoretical portfolio risk. The figure shows that the mean absolute risk error decreases as the number of assets increases under both Gaussian and Student-t return distributions. This indicates that portfolio risk can be estimated more accurately in higher-dimensional settings, reflecting the stabilizing effect of diversification. The error remains higher under the Student-t distribution, reflecting the greater uncertainty associated with heavy-tailed returns and the higher probability of extreme observations. These findings are consistent with [45], who emphasize the importance of estimation uncertainty in portfolio risk measurement and optimization.

**Figure 7:**
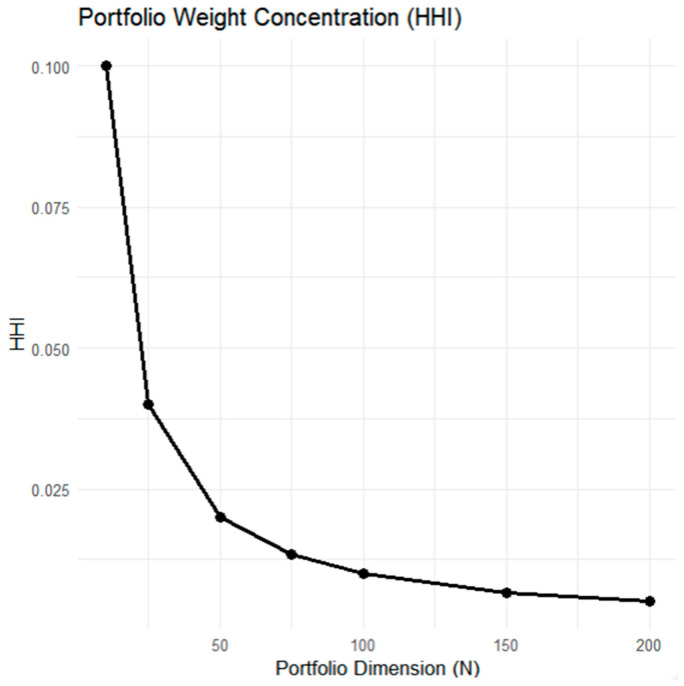
Evolution of the Herfindahl–Hirschman Index with portfolio dimension.

HHI decreases steadily as portfolio dimension increases, indicating lower weight concentration and improved diversification (Figure 7). As the number of assets grows, portfolio weights become more evenly distributed, and the influence of individual assets on overall portfolio performance diminishes. This reduction in concentration risk is consistent with the diversification principle of modern portfolio theory and suggests that larger portfolios achieve a more balanced allocation structure.

The empirical results can be interpreted along several dimensions. First, a rapid decline in tail probabilities indicates that portfolio returns become concentrated around their expected value as the portfolio dimension increases. Second, the decline in both portfolio risk and the standard deviation of realized risk suggests that larger portfolios exhibit higher stability and predictability. Third, risk estimation errors decrease with portfolio size, indicating improved reliability of risk assessment through diversification. Comparisons between Gaussian and Student-t return distributions show that heavy-tailed returns generate higher risk, greater variability, and weaker concentration effects. Finally, the estimated concentration parameter obtained from the log-tail regression provides an empirical measure of the exponential decay rate of tail probabilities, capturing the strength of concentration in the form $exp(-c\varepsilon^{2})$.

### 4.2. Distributional Robustness, Diversification, and Concentration Properties

**Figure 8:**
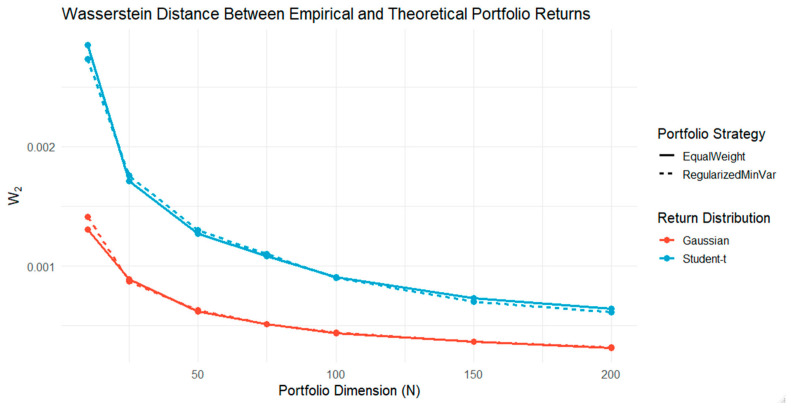
Wasserstein distance between empirical and theoretical portfolio returns across dimensions.

To assess distributional robustness, diversification, and concentration properties of portfolio returns, we examine three complementary measures: the Wasserstein distance, entropy, and Talagrand-type concentration bounds. The Wasserstein distance is a metric grounded in optimal transport theory that captures differences between probability distributions [46]. Table 5 and Figure 8 report the Wasserstein distances between empirical and theoretical portfolio return distributions under both Gaussian and Student-t return assumptions. The results reveal a decline in the Wasserstein distance as portfolio dimension increases, indicating that empirical portfolio returns become progressively closer to their theoretical counterparts in higher dimensions. Across all portfolio sizes, the Student-t distribution exhibits larger Wasserstein distances than the Gaussian distribution, reflecting the influence of heavier tails and a greater likelihood of extreme observations. Nevertheless, the distances remain relatively small in both cases, suggesting a close alignment between simulated portfolio returns and the assumed theoretical distributions.

The entropy increases with the number of assets, reflecting the larger support of the portfolio allocation (see Table 6). However, the normalized entropy remains equal to one for all values of N, indicating that the portfolio achieves maximum diversification in every case. This result is consistent with the Equal Weight structure, where all assets receive identical weights. Since entropy depends only on portfolio weights, Gaussian and Student-t return distributions generate identical entropy values. Consequently, diversification patterns are determined by portfolio dimension rather than by the assumed return distribution.

The Talagrand concentration inequality [20] is a fundamental result in probability theory that provides an upper bound on the probability that a random variable deviates from its mean. Table 7 summarizes the estimated concentration parameter and the corresponding Talagrand upper bound derived from the transport–entropy inequality. These quantities are theoretical characteristics of the concentration analysis and should not be confused with the Talagrand Stability Index (TSI), which is introduced separately in Section 3.5. The concentration parameter increases with portfolio dimension for both distributions, indicating stronger concentration of portfolio returns as the number of assets grows (see Table 7). However, concentration effects are stronger under the Gaussian distribution, which exhibits much larger values of c and considerably smaller Talagrand bounds. In contrast, the Student-t distribution generates weaker concentration and larger bounds due to its heavier tails and higher probability of extreme returns. The nearly identical estimates obtained for the Equal Weight and Regularized Minimum Variance portfolios suggest that portfolio dimension and the return distribution are the main determinants of concentration behavior.

The Talagrand bound is obtained by exponentiating the fitted intercept from the tail-probability regression, so it is highly sensitive to small fluctuations in that intercept. A modest shift in the intercept translates into a much larger apparent change in the bound. This explains the locally elevated bound observed for the Student-t case at N = 75, which reflects ordinary Monte Carlo variability in the regression fit rather than a discontinuity in the underlying concentration behavior.

### 4.3. Informational Dependence and Portfolio Diversification

**Figure 9:**
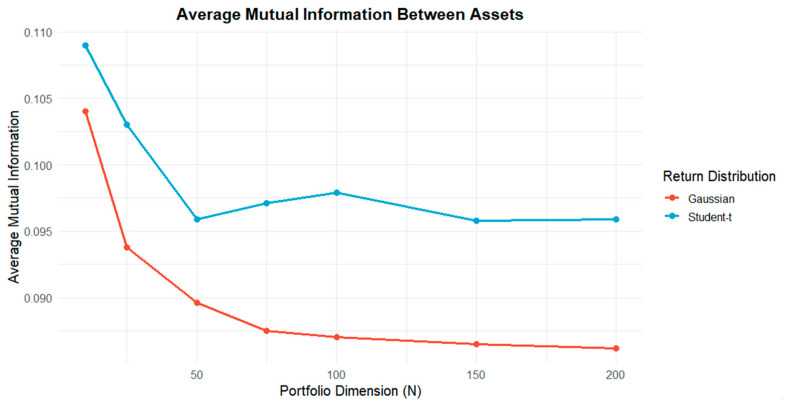
Average mutual information between assets as a function of portfolio size.

Ref. [14] shows that entropy- and mutual information-based portfolio frameworks can outperform traditional mean–variance approaches by better capturing nonlinear dependence structures and improving diversification and stability. Consistent with this perspective, Table 8 and Figure 9 illustrate the evolution of the average mutual information (MI) between asset returns as the portfolio dimension increases.

Average MI declines as portfolio dimension increases in both cases, indicating a reduction in informational overlap among assets and better diversification (Table 8). The Student-t distribution exhibits higher mutual information values than the Gaussian distribution, suggesting stronger dependence structures associated with heavy-tailed returns. However, the decline becomes progressively smaller for large portfolios, indicating diminishing marginal diversification benefits as additional assets are included.

Figure 9 shows that the average MI decreases with portfolio dimension under both Gaussian and Student-t return distributions. This indicates lower informational overlap and improved diversification in larger portfolios. The higher values observed under the Student-t distribution reflect stronger dependence induced by heavy-tailed returns, while the flattening of both curves suggests diminishing marginal diversification benefits as N increases.

### 4.4. Network-Based Representation of Dependence

To further investigate the structural organization of dependence, we construct financial networks based on MI computed from the simulated return series. Assets are represented as nodes, while links correspond to pairwise informational dependencies. Only the strongest dependence relationships, corresponding to the upper decile of the MI distribution, are retained through a quantile-based threshold.

**Figure 10:**
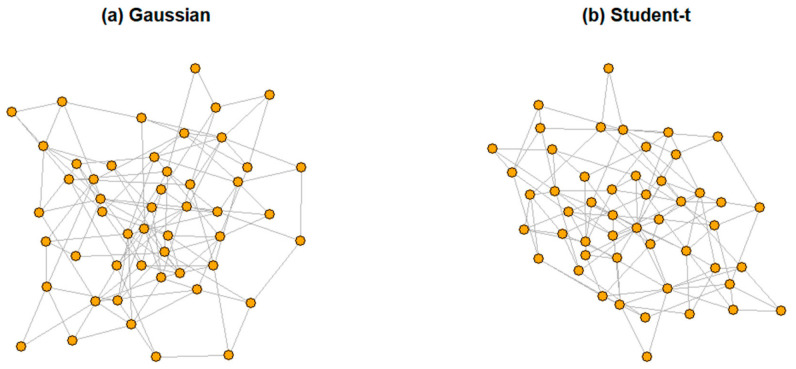
Financial dependence network based on mutual information among asset returns.

Figure 10 presents the resulting networks under Gaussian and Student-t return distributions. In both cases, the networks exhibit a moderately connected structure with several central nodes acting as informational hubs. These nodes are more strongly connected to the rest of the system and may therefore contribute to the transmission of shocks and systemic risk. The presence of peripheral nodes indicates weaker informational links and potential diversification opportunities.

The network statistics reported in Table 9 reveal similar topological properties across the two distributions. The density of approximately 0.10 indicates a sparse network in which only about 10% of all possible connections are present. The average degree of 4.92 suggests moderate interconnectedness, with each asset connected to roughly five other assets on average. The clustering coefficients are relatively low (0.1316 for Gaussian and 0.1349 for Student-t), indicating limited local clustering and the absence of highly cohesive asset groups. The average betweenness values are close across distributions, suggesting comparable patterns of information transmission within the network.

To assess the robustness of the mutual information network analysis, Table 9 reports the network characteristics obtained using filtering thresholds corresponding to the 80th, 85th, 90th, and 95th percentiles of the mutual information distribution. As expected, lower thresholds produce denser networks by retaining more dependence relationships. For example, reducing the threshold from the 90th to the 80th percentile increases network density from 0.1004 to 0.2000 and the average degree from 4.92 to 9.80. The clustering coefficient also increases, whereas the average betweenness centrality decreases because additional edges create alternative shortest paths. Conversely, increasing the threshold to the 95th percentile yields sparser networks with lower density, lower clustering, and higher betweenness centrality. Despite these quantitative changes, the qualitative comparison between the Gaussian and Student-t networks remains unchanged across all thresholds. The main conclusions are robust to the choice of the filtering threshold.

The MI networks obtained under the Gaussian and Student-t distributions in Figure 10 exhibit similar topological structures. Both networks are sparse and moderately connected, indicating that only the strongest nonlinear dependence relationships are retained. However, the Student-t network appears to display slightly stronger local clustering, suggesting that heavy-tailed returns generate more cohesive groups of interconnected assets. This pattern remains qualitatively unchanged when a less restrictive filtering threshold is applied, although both networks become denser and more interconnected as additional MI links are retained.

The Talagrand Stability Index (TSI) combines the Wasserstein distance and Kullback–Leibler divergence into a single measure of distributional stability. Lower TSI values indicate a closer agreement between empirical and theoretical portfolio return distributions and therefore greater portfolio stability.

**Figure 11:**
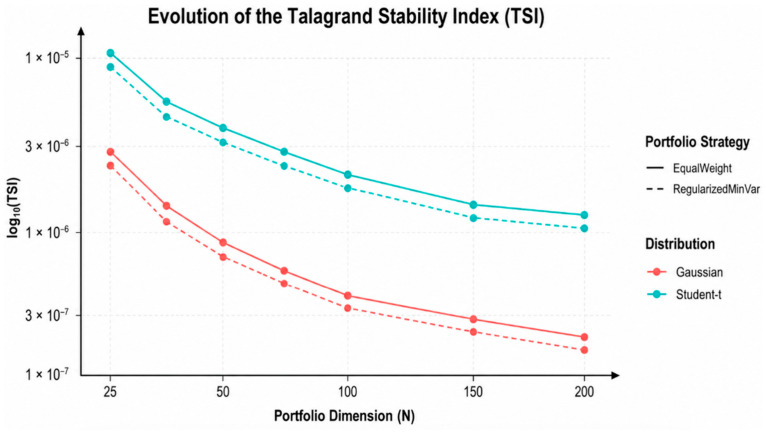
Evolution of the Talagrand Stability Index (TSI) across portfolio dimensions.

As shown in Table 10 and Figure 11, the TSI decreases systematically as portfolio dimension increases under both Gaussian and Student-t return distributions. This downward trend suggests that larger portfolios exhibit greater stability and stronger convergence between empirical and theoretical return behavior, reflecting the benefits of diversification.

A clear distinction emerges between the two return-generating processes. For all portfolio dimensions, the Student-t distribution produces larger TSI values than the Gaussian distribution. This result reflects the influence of heavy tails, which increase the likelihood of extreme observations and reduce distributional stability. Although diversification improves stability in both cases, convergence occurs more slowly under Student-t returns.

The differences between the Equal Weight and Regularized Minimum Variance portfolios are relatively small compared with the differences between the two distributions. This suggests that the return-generating process plays a more important role in determining stability than the choice of portfolio allocation rule.

### 4.5. Empirical Application of the Talagrand Stability Index

To provide a real-world illustration of the proposed framework, we analyze daily returns of ten large U.S. equities over the period 1 January 2020–31 December 2025, extracted from Yahoo Finance. The sample consists of Apple (AAPL), Microsoft (MSFT), JPMorgan Chase (JPM), Goldman Sachs (GS), Visa (V), Walmart (WMT), Coca-Cola (KO), McDonald’s (MCD), IBM (IBM), and Walt Disney (DIS). These firms represent several sectors of the U.S. economy, including technology, financial services, consumer goods, retail, and entertainment, thereby providing a diversified portfolio universe for empirical validation.

Equal Weight and Regularized Minimum Variance portfolios were constructed from these assets, and the Wasserstein distance, Kullback–Leibler divergence, and Talagrand Stability Index (TSI) were computed using daily log returns in Table 11.

For the empirical application, the reference distribution was chosen as the Gaussian distribution, parametrized by the empirical portfolio mean and empirical portfolio standard deviation. This choice provides a simple benchmark for comparing the empirical portfolio return distribution with its theoretical approximation.

The results indicate that both portfolio strategies exhibit small Wasserstein distances, suggesting a close alignment between empirical portfolio returns and their theoretical approximations. Similarly, the Kullback–Leibler divergences remain moderate, indicating limited informational divergence between empirical and theoretical return distributions.

A difference emerges in the TSI values. The Regularized Minimum Variance portfolio achieves a lower TSI (4.41 × 10^−5^) than the Equal Weight portfolio (6.99 × 10^−5^). Since lower TSI values correspond to greater distributional stability, this result suggests that the Regularized Minimum Variance strategy produces a more robust return distribution under actual market conditions. A lower TSI is associated with both a smaller Wasserstein distance and a lower KL divergence.

**Figure 12:**
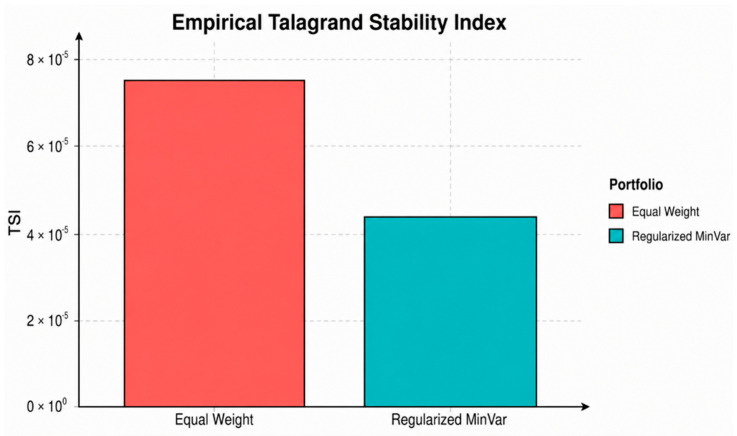
Empirical Talagrand Stability Index across portfolio strategies.

Figure 12 provides a graphical illustration of the empirical TSI for the two portfolio strategies. The lower bar associated with the Regularized Minimum Variance portfolio highlights its greater distributional stability relative to the Equal Weight portfolio. Although the difference is not large in absolute terms, it is consistent with the smaller Wasserstein distance, lower KL divergence, and lower TSI reported in Table 11.

To further assess the robustness of these findings, Appendix A extends the empirical analysis to a larger N = 50 asset universe, reporting rolling-window estimates, sensitivity analyses, and out-of-sample performance metrics for both portfolio strategies.

## 5. Conclusions

This paper examines how portfolio dimensionality affects financial stability using concentration-of-measure theory, information-theoretic metrics, and transport-information inequalities. We introduce the Talagrand Stability Index (TSI), a new measure that combines the Wasserstein distance and the Kullback–Leibler divergence to assess distributional stability. Using simulated portfolio returns under Gaussian and Student-t distributions, we analyze how diversification, concentration, and stability change as the number of assets increases.

The TSI results support the central hypothesis that larger portfolios are more stable due to stronger concentration effects, better diversification, and greater robustness. For both Gaussian and Student-t returns, the Wasserstein distance and Kullback–Leibler divergence remain low, indicating close agreement between empirical and theoretical distributions. TSI declines as portfolio dimension increases, showing that distributional discrepancies become less important in larger portfolios.

These findings are consistent with the concentration-of-measure results, which show lower portfolio risk, smaller estimation errors, stronger concentration, and reduced tail probabilities as the number of assets grows. Information-theoretic measures prove that higher entropy reflects greater diversification, while lower mutual information and sparse financial networks indicate weaker dependence among assets.

Comparing Gaussian and Student-t distributions highlights the role of tail behavior. Although diversification improves stability in both cases, Student-t returns exhibit weaker concentration, stronger dependence, larger Talagrand bounds, and higher TSI values. Thus, heavy-tailed distributions remain associated with greater uncertainty and lower stability, even in high-dimensional portfolios.

An important implication is that portfolio dimension is a main driver of stability. The small differences between Equal Weight and Regularized Minimum Variance portfolios suggest that, under the symmetric covariance structure considered, diversification contributes more to stability than the specific allocation rule.

Methodologically, the TSI is a novel contribution that combines geometric and information-theoretic measures into a single indicator of distributional stability. Unlike traditional risk measures focused on volatility, it provides a broader assessment of portfolio behavior and can complement existing risk management tools.

At the same time, robustness checks reported in Appendix A show that TSI’s incremental value is outcome-dependent rather than uniform. TSI is highly correlated with its own Wasserstein-distance component (Table A7), is not a statistically significant predictor of realized volatility or short-horizon tail probability once the Wasserstein distance, KL divergence, and portfolio-weight entropy are accounted for, and adds significant explanatory power only for maximum drawdown (Table A8). Likewise, the bootstrap confidence intervals for out-of-sample TSI overlap substantially between the Equal Weight and Regularized Minimum Variance portfolios (Table A6), so portfolio-level differences in TSI should be read as suggestive rather than decisive. We therefore view TSI as a complementary, outcome-dependent indicator of distributional stability rather than a generally superior standalone risk measure.

Investors should prioritize broad diversification, as increasing portfolio dimensionality improves stability, reduces concentration risk, and enhances robustness.

The analysis is based on simulated returns with controlled covariance structures and homogeneous expected returns. While this framework allows the examination of diversification and stability, it does not capture all features of real markets, such as regime shifts, heterogeneous assets, market frictions, and time-varying dependence. The study is also limited to two portfolio allocation strategies.

Future research could apply the framework to real financial datasets, including equity, fixed-income, cryptocurrency, and ESG portfolios, to validate the TSI under realistic conditions. Further work may also explore alternative dependence structures, dynamic covariance models, and more advanced portfolio allocation methods.

## Figures and Tables

**Table 1:** Portfolio risk, stability, and diversification.

Distribution	N	Avg. Risk	SD Risk	Risk Error	HHI
Gaussian	10	0.00920	0.000422	0.000340	0.1000
Gaussian	25	0.00600	0.000262	0.000210	0.0400
Gaussian	50	0.00427	0.000188	0.000144	0.0200
Gaussian	75	0.00350	0.000161	0.000120	0.0133
Gaussian	100	0.00302	0.000143	0.000120	0.0100
Gaussian	150	0.00249	0.000109	0.000086	0.0067
Gaussian	200	0.00216	0.000100	0.000077	0.0050
Student-t	10	0.01180	0.000899	0.000701	0.1000
Student-t	25	0.00770	0.000575	0.000464	0.0400
Student-t	50	0.00552	0.000451	0.000339	0.0200
Student-t	75	0.00452	0.000353	0.000264	0.0133
Student-t	100	0.00388	0.000279	0.000221	0.0100
Student-t	150	0.00320	0.000228	0.000180	0.0067
Student-t	200	0.00275	0.000211	0.000170	0.0050

**Table 2:** Concentration parameter *c* and intercept estimates.

Distribution	N	Portfolio	Concentration Parameter (c)	Intercept
Gaussian	10	Equal Weight	0.6760	−0.5671
Gaussian	10	Reg. Min Variance	0.6759	−0.5678
Gaussian	25	Equal Weight	1.3731	−0.8466
Gaussian	25	Reg. Min Variance	1.3731	−0.8469
Gaussian	50	Equal Weight	3.2511	−0.5234
Gaussian	50	Reg. Min Variance	3.2514	−0.5239
Gaussian	75	Equal Weight	4.8453	−0.5110
Gaussian	75	Reg. Min Variance	4.8455	−0.5095
Gaussian	100	Equal Weight	6.3867	−0.5409
Gaussian	100	Reg. Min Variance	6.3851	−0.5399
Gaussian	150	Equal Weight	9.5310	−0.5195
Gaussian	150	Reg. Min Variance	9.5311	−0.5202
Gaussian	200	Equal Weight	11.5040	−0.6964
Gaussian	200	Reg. Min Variance	11.5033	−0.6969
Student-t	10	Equal Weight	0.1172	−2.0490
Student-t	10	Reg. Min Variance	0.1169	−2.0532
Student-t	25	Equal Weight	0.1370	−3.1877
Student-t	25	Reg. Min Variance	0.1370	−3.1873
Student-t	50	Equal Weight	0.1799	−3.7414
Student-t	50	Reg. Min Variance	0.1797	−3.7372
Student-t	75	Equal Weight	0.1834	−4.5655
Student-t	75	Reg. Min Variance	0.1835	−4.5632
Student-t	100	Equal Weight	0.4459	−3.6384
Student-t	100	Reg. Min Variance	0.4459	−3.6356
Student-t	150	Equal Weight	0.7505	−3.1863
Student-t	150	Reg. Min Variance	0.7504	−3.1866
Student-t	200	Equal Weight	0.7984	−3.7713
Student-t	200	Reg. Min Variance	0.8137	−3.7689

**Table 3:** Linear relationship between portfolio dimension and concentration parameter—Gaussian distribution.

Variable	Coefficient	Std. Error	t-Stat	*p*-Value
Intercept	0.228	0.157	1.45	0.172
N	0.059	0.0015	40.48	<0.001

**Table 4:** Linear relationship between portfolio dimension and concentration parameter—Student-t distribution.

Variable	Coefficient	Std. Error	t-Stat	*p*-Value
Intercept	0.013	0.0584	0.22	0.834
N	0.004	0.0005	7.63	<0.001

**Table 5:** Wasserstein distance between empirical and theoretical portfolio returns.

Distribution	N	Wasserstein Distance
Gaussian	10	0.00131
Gaussian	25	0.00089
Gaussian	50	0.00062
Gaussian	75	0.00051
Gaussian	100	0.00044
Gaussian	150	0.00036
Gaussian	200	0.00032
Student-t	10	0.00285
Student-t	25	0.00171
Student-t	50	0.00127
Student-t	75	0.00109
Student-t	100	0.00091
Student-t	150	0.00073
Student-t	200	0.00064

**Table 6:** Entropy of portfolio weights across dimensions.

Number of Assets (N)	Entropy (H(w))	Normalized Entropy
10	2.3026	1.0000
25	3.2189	1.0000
50	3.9120	1.0000
75	4.3175	1.0000
100	4.6052	1.0000
150	5.0106	1.0000
200	5.2983	1.0000

**Table 7:** Talagrand-type concentration bounds across portfolio dimensions.

Distribution	N	c (Equal Weight)	c (Reg. Min Var)	Intercept (EW)	Intercept (RMV)	Talagrand Bound
Gaussian	10	0.676	0.676	−0.567	−0.568	1.76
Gaussian	25	1.370	1.370	−0.847	−0.847	2.33
Gaussian	50	3.250	3.250	−0.523	−0.524	1.69
Gaussian	75	4.850	4.850	−0.511	−0.509	1.67
Gaussian	100	6.390	6.390	−0.541	−0.540	1.72
Gaussian	150	9.530	9.530	−0.520	−0.520	1.68
Gaussian	200	11.500	11.500	−0.696	−0.697	2.01
Student-t	10	0.117	0.117	−2.05	−2.05	7.78
Student-t	25	0.137	0.137	−3.19	−3.19	24.2
Student-t	50	0.180	0.180	−3.74	−3.74	42.1
Student-t	75	0.183	0.184	−4.57	−4.56	96.0
Student-t	100	0.446	0.446	−3.64	−3.64	38.0
Student-t	150	0.750	0.750	−3.19	−3.19	24.2
Student-t	200	0.798	0.814	−3.77	−3.77	43.4

**Table 8:** Average mutual information between assets across portfolio dimensions.

Number of Assets (N)	Mutual Information (Gaussian)	Mutual Information (Student-t)
10	0.1040	0.1090
25	0.0938	0.1030
50	0.0896	0.0959
75	0.0875	0.0971
100	0.0870	0.0979
150	0.0865	0.0958
200	0.0862	0.0959

**Table 9:** Topological characteristics of mutual information financial networks under alternative thresholds.

Threshold	Distribution	Density	Average Degree	Clustering Coefficient	Average Betweenness
95%	Gaussian	0.0506	2.48	0.0273	91.42
95%	Student-t	0.0506	2.48	0.1008	120.26
90%	Gaussian	0.1004	4.92	0.1316	38.84
90%	Student-t	0.1004	4.92	0.1349	39.52
85%	Gaussian	0.1502	7.36	0.1785	28.28
85%	Student-t	0.1502	7.36	0.1740	28.40
80%	Gaussian	0.2000	9.80	0.1956	22.14
80%	Student-t	0.2000	9.80	0.2379	22.50

**Table 10:** Talagrand Stability Index (TSI) across portfolio dimensions.

Distribution	N	Equal Weight	Regularized Minimum Variance
Gaussian	10	3.85 × 10^−6^	3.91 × 10^−6^
Gaussian	25	1.65 × 10^−6^	1.55 × 10^−6^
Gaussian	50	7.10 × 10^−7^	8.05 × 10^−7^
Gaussian	75	5.36 × 10^−7^	5.50 × 10^−7^
Gaussian	100	3.67 × 10^−7^	3.92 × 10^−7^
Gaussian	150	2.81 × 10^−7^	2.69 × 10^−7^
Gaussian	200	2.17 × 10^−7^	2.05 × 10^−7^
Student-t	10	2.10 × 10^−5^	1.91 × 10^−5^
Student-t	25	1.00 × 10^−5^	8.07 × 10^−6^
Student-t	50	5.66 × 10^−6^	4.81 × 10^−6^
Student-t	75	3.57 × 10^−6^	3.15 × 10^−6^
Student-t	100	2.47 × 10^−6^	2.21 × 10^−6^
Student-t	150	1.49 × 10^−6^	1.38 × 10^−6^
Student-t	200	1.33 × 10^−6^	1.03 × 10^−6^

**Table 11:** Empirical Talagrand Stability Index for U.S. equity portfolios.

Portfolio Strategy	Wasserstein Distance	KL Divergence	Talagrand Stability Index (TSI)
Equal Weight	0.00551	0.4344	6.99 × 10^−5^
Regularized Minimum Variance	0.00409	0.3795	4.41 × 10^−5^

**Figure A1:**
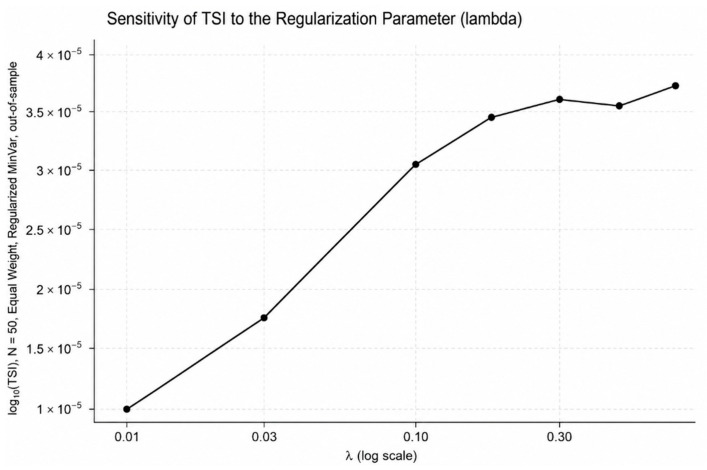
Sensitivity of out-of-sample TSI (Regularized Minimum Variance, N = 50) to the regularization parameter λ (log scale).

**Figure A2:**
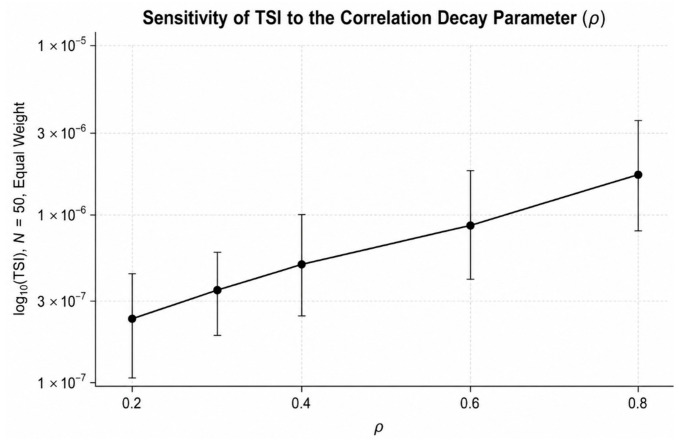
Sensitivity of simulated TSI (N = 50, Equal Weight, Gaussian design) to the correlation decay parameter ρ; error bars show ±1 Monte Carlo standard deviation across 100 replications (log scale).

**Figure A3:**
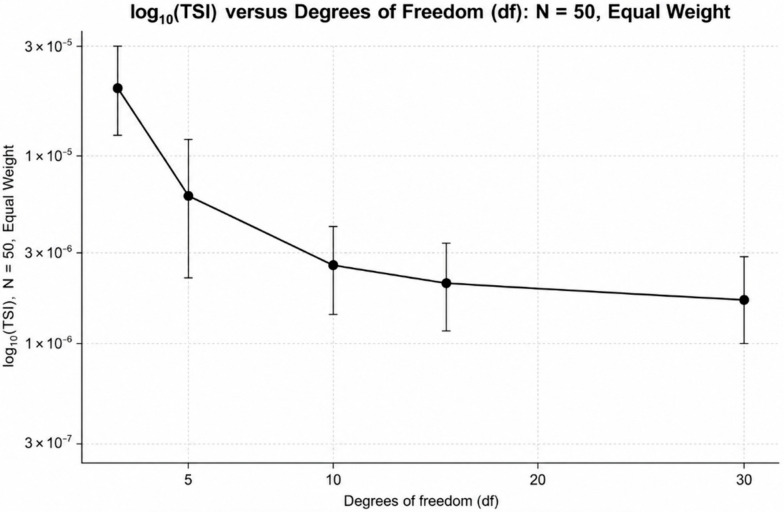
Sensitivity of simulated TSI (N = 50, Equal Weight) to the Student-t degrees of freedom ν; error bars show ±1 Monte Carlo standard deviation across 100 replications (log scale).

**Figure A4:**
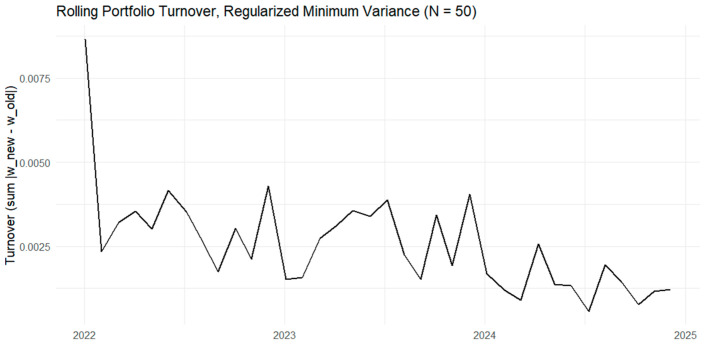
Rolling monthly portfolio turnover, Regularized Minimum Variance portfolio (N = 50), 2022–2025.

**Figure A5:**
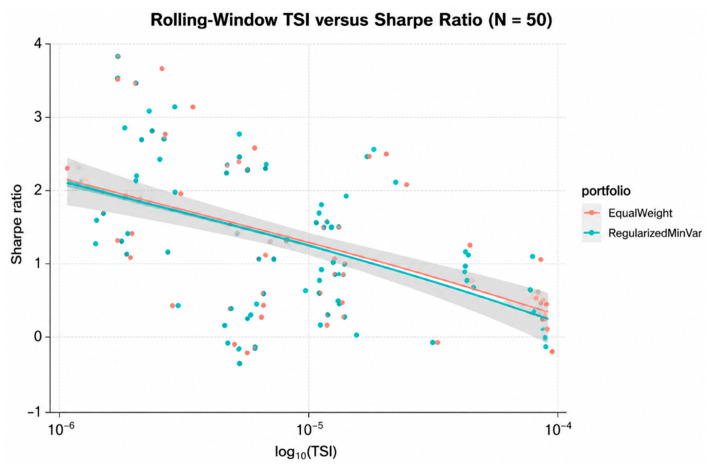
Rolling-window TSI versus rolling-window Sharpe ratio, N = 50, with fitted linear trends and 95% confidence bands by portfolio.

## Data Availability

Data is available upon request.

## Appendix A

To support the paper’s high-dimensional framing, this appendix extends the real-data application from ten to fifty sector-diversified U.S. equities of the S&P 500, spanning technology, financial services, energy, healthcare, consumer staples, discretionary, communication services, and industrials (see Table A1). The training (in-sample) window spans 1 January 2016 to 1 January 2022, and the test (out-of-sample) window spans 1 January 2022 to 31 December 2025, giving a return matrix of approximately 1512 trading days by 50 assets for training and approximately 1006 trading days by 50 assets for testing.

**Table A1:** Composition of the extended N = 50 asset universe, by sector.

Sector	Tickers
Technology	AAPL, MSFT, NVDA, AMZN, GOOGL, META, AVGO, ORCL, CRM, ADBE, IBM
Financials	JPM, BAC, GS, MS, V, MA, AXP, BLK, SCHW, C
Energy	XOM, CVX, COP, SLB, EOG
Healthcare	JNJ, PFE, UNH, MRK, ABBV, LLY
Consumer Staples/Discretionary	PG, KO, PEP, WMT, COST, MCD, NKE, HD, LOW
Communication Services	DIS, CMCSA, T, VZ
Industrials	CAT, BA, HON, GE, UPS

Let $R_{p,t}$ denote the realized portfolio return on day t, let T denote the number of return observations in the relevant window, and let ${\bar{R}}_{p}=\frac{1}{T}\sum_{t=1}^{T}R_{p,t}$ denote the sample mean return. The annualized Sharpe ratio is defined as:

(A1) $$SR=\frac{{\bar{R}}_{p}-r_{f}}{{\hat{\sigma }}_{p}}\sqrt{252},\;\;{\hat{\sigma }}_{p}=\sqrt{\frac{1}{T-1}\sum_{t=1}^{T}{\left(R_{p,t}-{\bar{R}}_{p}\right)}^{2}}$$

where $r_{f}$ denotes the risk-free rate (set to zero in this appendix, consistent with the daily return, short-horizon setting of Section 3) and the factor $\sqrt{252}$ annualizes the daily ratio. The measure was introduced by [47] to evaluate mutual fund performance, and was later formalized by [48].

Let $V_{t}=\prod_{s=1}^{t}\left(1+R_{p,s}\right)$ denote the cumulative wealth index implied by the portfolio return series, normalized to $V_{0}=1$. The maximum drawdown over the window is:

(A2) $$MDD=\underset{1\leq t\leq T}{min}\left(\frac{V_{t}}{\underset{0\leq s\leq t}{max}V_{s}}-1\right)\leq 0,$$

i.e., the largest peak-to-trough percentage decline in cumulative wealth observed over the evaluation window. Drawdown-based risk measures and their use as portfolio-optimization constraints were formalized by [49], and their statistical properties (distribution, expected value, and estimation) were later analyzed by [50].

Value-at-Risk was popularized as an industry risk-management standard by J.P. Morgan’s RiskMetrics methodology [51] and formally surveyed by [52,53].

The α-level historical (empirical) Value-at-Risk is the empirical α-quantile of the realized return distribution

(A3) $$VaR_{\alpha }=inf\left\{r\in R:\hat{F}\left(r\right)\geq \alpha \right\},$$

where $\hat{F}$ denotes the empirical cumulative distribution function of $\{R_{p,t}{\}}_{t=1}^{T}$ and α=0.05 throughout this appendix.

Also known as Expected Shortfall, the α-level Conditional Value-at-Risk is the empirical mean return conditional on being at or below the corresponding VaR threshold

(A4) CVaRα=ERp∣Rp≤VaRα≈1αT∑t: Rp,t≤VaRαRp,t

where T is the sample size, α is the tail probability, $\left\lceil \alpha T\right\rceil$ is the number of observations in the lower tail.

CVaR was introduced by [54] and was developed into a convex optimization framework for portfolio choice by [55].

For a portfolio rebalanced from weights $w_{old}$ to $w_{new}$, one-way turnover is defined as:

(A5) $$Turnover=\sum_{i=1}^{N}\left|w_{i,new}-w_{i,old}\right|$$

Average turnover is the mean of this quantity across monthly rebalancing dates in the out-of-sample rolling re-estimation. Portfolio turnover as a proxy for transaction costs in the evaluation of minimum-variance and other data-driven allocation rules was used by [30], whose framework is followed here.

Table A2 reports, for both the Equal Weight and the Regularized Minimum Variance portfolios and for both the in-sample and out-of-sample windows, the realized mean and standard deviation of portfolio returns, the Sharpe ratio [48], the maximum drawdown, the 5% Value-at-Risk and Conditional Value-at-Risk [55], the estimated concentration parameter $\hat{c}$, the Wasserstein-2 distance, the Kullback–Leibler divergence, and TSI.

**Table A2:** In-sample versus out-of-sample portfolio statistics, N = 50.

Portfolio	Sample	Mean	SD	Sharpe	Max Drawdown	VaR (5%)	CVaR (5%)	$\hat{c}$	Wasserstein	KL	TSI
Equal Weight	In−sample	0.000648	0.012342	0.833	−0.3897	−0.0172	−0.0311	0.0729	0.005738	0.33787	9.830 × 10^−5^
Equal Weight	Out−of−sample	0.000418	0.010182	0.651	−0.2380	−0.0156	−0.0241	0.1014	0.002725	0.24722	3.082 × 10^−5^
Regularized MinVar	In−sample	0.000648	0.012261	0.839	−0.3867	−0.0171	−0.0309	0.0727	0.005708	0.34143	9.633 × 10^−5^
Regularized MinVar	Out−of−sample	0.000414	0.010124	0.649	−0.2372	−0.0154	−0.0239	0.1093	0.002705	0.24612	3.049 × 10^−5^

For both portfolios, out-of-sample TSI is about three times smaller than in-sample TSI, driven mainly by a decline in the Wasserstein distance rather than in the KL divergence. Sharpe ratios and realized risk are similar in-sample. The Regularized Minimum Variance portfolio attains a marginally lower out-of-sample TSI than the Equal Weight portfolio (3.049 × 10^−5^ versus 3.082 × 10^−5^), despite a marginally lower out-of-sample Sharpe ratio. As Table A6 shows, the bootstrap distributions of TSI for the two portfolios overlap substantially, so this difference should be read as a slight, not a pronounced or statistically decisive, advantage. The concentration parameter $\hat{c}$ is larger out-of-sample than in-sample for both portfolios.

The robustness of the results is assessed with respect to three parameters: the shrinkage (regularization) parameter λ used in the Regularized Minimum Variance portfolio [31,32], the correlation decay parameter ρ of the Toeplitz covariance structure, and the degrees of freedom ν of the Student-t data-generating process. For λ, sensitivity is assessed directly on the real out-of-sample return data; for ρ and ν, sensitivity is assessed via Monte Carlo simulation at N = 50. Realized risk increases monotonically, and then flattens, as λ increases from 0.01 to 0.80. HHI decreases monotonically over the same range, TSI, by contrast, varies only mildly with λ and remains confined to a comparatively narrow range (2.90 × 10^−5^ to 3.07 × 10^−5^), rising from λ = 0.01 to λ = 0.20, dips slightly at λ = 0.40. It indicates limited overall sensitivity to the regularization parameter (see Table A3).

**Table A3:** Sensitivity of out-of-sample portfolio statistics to the regularization parameter λ (Regularized Minimum Variance, N = 50.

λ	SD (Realized)	HHI	$\hat{c}$	Wasserstein	KL	TSI
0.01	0.009682	0.020767	0.118111	0.002549	0.230156	2.899 × 10^−5^
0.05	0.010067	0.020039	0.109058	0.002686	0.245097	3.016 × 10^−5^
0.10	0.010124	0.020010	0.109307	0.002705	0.246121	3.049 × 10^−5^
0.20	0.010153	0.020003	0.101549	0.002715	0.247030	3.061 × 10^−5^
0.40	0.010167	0.020001	0.101580	0.002720	0.248373	3.055 × 10^−5^
0.80	0.010175	0.020000	0.101328	0.002723	0.248093	3.065 × 10^−5^

Figure A1 shows that TSI changes only modestly across the values of λ considered. It reaches its minimum at λ = 0.01, rises to a local peak at λ = 0.20, dips at λ = 0.40, and rises again at λ = 0.80. Overall, the differences are small, indicating limited sensitivity to the regularization parameter. This supports the use of λ = 0.10 as a reasonable choice.

Table A4 indicates that TSI increases with the correlation parameter ρ, suggesting that stronger dependence among assets reduces portfolio stability. The value ρ = 0.4 adopted in the main simulations corresponds to a moderate dependence scenario.

**Table A4:** Sensitivity of the simulated Talagrand Stability Index to the correlation decay parameter ρ (N = 50, Equal Weight, Gaussian design, 100 replications).

ρ	Mean TSI	SD of TSI
0.10	3.196 × 10^−7^	8.069 × 10^−8^
0.25	4.295 × 10^−7^	1.144 × 10^−7^
0.40	6.017 × 10^−7^	1.605 × 10^−7^
0.55	8.738 × 10^−7^	2.244 × 10^−7^
0.70	1.3257 × 10^−6^	3.834 × 10^−7^

Figure A2 shows that the mean TSI increases as the correlation parameter ρ increases. The wider error bars at higher values of ρ indicate greater Monte Carlo variability, reflecting the stronger sensitivity of portfolio stability to highly correlated asset returns.

Table A5 shows that TSI decreases as the degrees of freedom ν increase, indicating that heavier tails lead to larger deviations between the empirical and reference return distributions.

**Table A5:** Sensitivity of the simulated Talagrand Stability Index to the Student-t degrees of freedom ν (N = 50, Equal Weight, 100 replications).

ν	Mean TSI	SD of TSI
3	2.250 × 10^−5^	3.608 × 10^−5^
5	3.401 × 10^−6^	4.117 × 10^−6^
8	1.301 × 10^−6^	3.415 × 10^−7^
15	8.312 × 10^−7^	3.415 × 10^−7^
30	7.311 × 10^−7^	3.551 × 10^−7^

Figure A3 shows that the mean TSI decreases as the Student-t degrees of freedom (ν) increase, indicating that heavier-tailed return distributions produce larger deviations between the empirical and reference return distributions. The decline is steep for low values of ν and becomes more gradual as ν increases.

In total, 95% confidence intervals for the concentration parameter, the Wasserstein-2 distance, the Kullback–Leibler divergence, and TSI are obtained by a moving-block bootstrap (block length of 20 trading days, 500 bootstrap replications) applied to the out-of-sample return series of each portfolio in Table A6. The bootstrap distributions of TSI for the Equal Weight and Regularized Minimum Variance portfolios overlap substantially. A similar conclusion applies to the concentration parameter, Wasserstein distance, and KL divergence.

**Table A6:** Block-bootstrap 95% confidence intervals for distributional-stability statistics, out-of-sample, N = 50.

Portfolio	Statistic	Lower 95%	Median	Upper 95%
Equal Weight	$\hat{c}$	0.0772	0.1030	0.3932
Equal Weight	Wasserstein	0.001436	0.002683	0.004281
Equal Weight	KL	0.1820	0.2788	0.4068
Equal Weight	TSI	8.506 × 10^−6^	2.997 × 10^−5^	5.395 × 10^−5^
Regularized MinVar	$\hat{c}$	0.0796	0.1065	0.4287
Regularized MinVar	Wasserstein	0.001299	0.002679	0.004180
Regularized MinVar	KL	0.1889	0.2836	0.3802
Regularized MinVar	TSI	7.488 × 10^−6^	2.987 × 10^−5^	5.224 × 10^−5^

Table A7 reports the pairwise correlation matrix of realized risk, the tail probability of a 2% daily move, maximum drawdown, the Wasserstein distance, the KL divergence, TSI, the concentration parameter $\hat{c}$, and normalized portfolio-weight entropy, computed over the rolling-window panel described below. Table A7 confirms that TSI is influenced mainly by the Wasserstein distance rather than by the Kullback–Leibler divergence. TSI is almost perfectly correlated with the Wasserstein distance (r = 0.989), while its correlation with KL is weaker (r = 0.323). Changes in TSI across the rolling windows are driven mainly by changes in the Wasserstein distance. The KL divergence contributes less. The moderate negative correlations between normalized entropy and both the Wasserstein distance (r = −0.574) and TSI (r = −0.575) indicate that higher entropy is associated with lower TSI values and Wasserstein distances.

**Table A7:** Correlation matrix of distributional stability and risk measures, rolling windows, N = 50.

	Realized_sd	Tail_prob_2pct	Max_drawdown	Wasserstein	KL	TSI	c_hat	Entropy_norm
Realized_sd	1.000	0.937	−0.938	0.884	0.343	0.879	−0.608	−0.601
Tail_prob_2pct	0.937	1.000	−0.894	0.718	0.305	0.698	−0.617	−0.536
Max_drawdown	−0.938	−0.894	1.000	−0.859	−0.379	−0.837	0.604	0.537
Wasserstein	0.884	0.718	−0.859	1.000	0.346	0.989	−0.582	−0.574
KL	0.343	0.305	−0.379	0.346	1.000	0.323	0.049	−0.284
TSI	0.879	0.698	−0.837	0.989	0.323	1.000	−0.488	−0.575
c_hat	−0.608	−0.617	0.604	−0.582	0.049	−0.488	1.000	0.274
Entropy_norm	−0.601	−0.536	0.537	−0.574	−0.284	−0.575	0.274	1.000

To assess whether TSI conveys information beyond its own components (the Wasserstein distance and the Kullback–Leibler divergence) and beyond standard portfolio diversification measures, a rolling-window panel is constructed (250-day windows, stepped monthly; 216 pooled observations across both portfolios). The realized risk, the tail probability of a 2% daily move, and the maximum drawdown within each window are regressed on the Wasserstein distance, the Kullback–Leibler divergence, TSI, and normalized portfolio-weight entropy. A nested F-test compares this specification to a restricted model excluding TSI. The results are shown in Table A8.

For realized portfolio risk, TSI is not statistically significant (*p* = 0.268), and the nested F-test confirms that including TSI does not improve model fit (F(1,211) = 1.23, *p* = 0.268). A similar pattern is observed for the tail probability of a 2% loss, where TSI remains statistically insignificant at the 5% level (*p* = 0.076). In contrast, TSI is a significant predictor of maximum drawdown at the 5% level (*p* = 0.030), indicating that it captures information relevant to cumulative downside risk beyond that contained in the Wasserstein distance, KL divergence, and normalized entropy. Across all three models, the Wasserstein distance is the most consistent predictor, whereas the KL divergence is not statistically significant in any specification. These results suggest that the incremental value of TSI is outcome-dependent, being most informative for explaining maximum drawdown rather than realized volatility or tail risk.

**Table A8:** Incremental-value regressions: realized risk, tail probability, and maximum drawdown on Wasserstein distance, KL divergence, TSI, and normalized entropy (rolling windows, N = 50).

Outcome	Term	Estimate	Std. Error	t-Statistic	*p*-Value
Realized SD	Intercept	3.58	1.03	3.47	<0.001
Realized SD	Wasserstein	0.993	0.372	2.67	0.008
Realized SD	KL	1.56 × 10^−3^	1.54 × 10^−3^	1.02	0.311
Realized SD	TSI	29.0	26.1	1.11	0.268
Realized SD	Entropy (norm.)	−3.58	1.03	−3.47	<0.001
Tail Prob (2%)	Intercept	57.4	17.6	3.26	0.001
Tail Prob (2%)	Wasserstein	23.1	6.33	3.65	<0.001
Tail Prob (2%)	KL	0.0177	0.0262	0.678	0.499
Tail Prob (2%)	TSI	−792	444	−1.78	0.076
Tail Prob (2%)	Entropy (norm.)	−57.4	17.6	−3.27	0.001
Max Drawdown	Intercept	−33.0	23.1	−1.43	0.154
Max Drawdown	Wasserstein	−46.4	8.33	−5.57	<0.001
Max Drawdown	KL	−0.0701	0.0344	−2.04	0.043
Max Drawdown	TSI	1276	584	2.19	0.030
Max Drawdown	Entropy (norm.)	33.1	23.1	1.43	0.154

Table A9 reports standard financial performance measures for the out-of-sample period, together with the average monthly turnover of the Regularized Minimum Variance portfolio under monthly rolling re-estimation. The out-of-sample financial performance of the Equal Weight and Regularized Minimum Variance portfolios for N = 50 assets are compared. Table A9 shows that the Equal Weight and Regularized Minimum Variance portfolios deliver very similar out-of-sample financial performance. Sharpe ratios and downside-risk measures differ only marginally, and the Regularized Minimum Variance portfolio attains a marginally lower TSI (3.049 × 10^−5^ versus 3.082 × 10^−5^). As Table A6 shows, this difference is well within the overlap of the two portfolios’ bootstrap confidence intervals, so it should be read as a slight rather than a decisive edge in distributional stability. This modest change is obtained with a low average monthly turnover of 0.25% of portfolio value.

**Table A9:** Out-of-sample financial performance, N = 50.

Portfolio	Sharpe	Max Drawdown	VaR (5%)	CVaR (5%)	TSI (Out-of-Sample)	Average Monthly Turnover
Equal Weight	0.6510	−0.2380	−0.01557	−0.02407	3.082 × 10^−5^	–
Regularized MinVar	0.6492	−0.2372	−0.01542	−0.02394	3.049 × 10^−5^	0.002537

Figure A4 shows a pronounced turnover spike at the first rebalancing date, followed by low turnover throughout the rolling evaluation period. This indicates that the Regularized Minimum Variance portfolio requires only modest portfolio adjustments over time.

Table A10 shows a moderate negative association between TSI and the rolling Sharpe ratio for both portfolio strategies. The Spearman correlations of −0.547 for the Equal Weight portfolio and −0.537 for the Regularized Minimum Variance portfolio indicate that periods with lower TSI values tend to coincide with higher risk-adjusted performance.

**Table A10:** Correlation between out-of-sample TSI and the Sharpe ratio across rolling windows, N = 50.

Portfolio	Correlation (TSI, Sharpe)
Equal Weight	−0.547
Regularized MinVar	−0.537

Figure A5 illustrates the relationship between rolling-window TSI and the corresponding Sharpe ratio for the two portfolio strategies. In both cases, the fitted trend is downward sloping, consistent with the moderate negative Spearman correlations reported in Table A10. Thus, periods with lower TSI values tend to coincide with higher risk-adjusted performance. Although the scatter around the fitted lines is pronounced, the negative association is similar for both the Equal Weight and Regularized Minimum Variance portfolios.
